# Longitudinal sepsis-induced coagulopathy score trajectories and mortality in relation to early prophylactic heparin: a multicentre cohort study

**DOI:** 10.3389/fcimb.2026.1923833

**Published:** 2026-09-24

**Authors:** Yuan Cao, Min Wang, Yun Li, Shaoqing Shi, Chengjin Wang, Yiqi Wu, Zhen Gao, Lu Wang, Yuyan Liu, Hongjun Kang

**Affiliations:** 1 Medical School of Chinese PLA, Beijing, China; 2 Department of Critical Care Medicine, the First Medical Centre, Chinese PLA General Hospital, Beijing, China; 3 Medical Intensive Care Unit, General Hospital of Southern Theater Command of PLA, Guangzhou, Guangzhou, China; 4 Department of Critical Care Medicine, Fifth Medical Centre of Chinese PLA General Hospital, Beijing, China; 5 Department of Critical Care Medicine, Chinese PAP Beijing Corps Hospital, Beijing, China

**Keywords:** dynamic trajectory, heparin therapy, latent class mixed model, sepsis, sepsis-induced coagulopathy

## Abstract

**Background:**

Sepsis-induced coagulopathy (SIC) evolves dynamically, and a single-timepoint score may not capture its longitudinal course. This study characterised SIC score trajectories, evaluated their associations with mortality, and conducted exploratory analyses of early prophylactic unfractionated heparin exposure.

**Methods:**

SIC score trajectories were characterised using a latent class mixed model. Trajectory-class prediction models were developed from baseline features within the first 24 hours of ICU admission using five algorithms. Heparin analysis used a 48-hour landmark design; weighted Fine–Gray models and cumulative incidence functions estimated 28-day post-landmark in-hospital mortality.

**Results:**

The study enrolled 20,168 patients from MIMIC-IV and 5,829 from eICU-CRD. Three SIC trajectories were identified—Persistently High, Rising-then-Falling, and Resolving—comprising 31.9%, 10.6%, and 57.5% of patients in MIMIC-IV and 49.1%, 7.2%, and 43.7% in eICU-CRD. Relative to the Resolving trajectory, adjusted subdistribution hazard ratios (sHR) for mortality were 1.65 (95% CI 1.52–1.78) and 1.33 (1.18–1.49) for Persistently High and Rising-then-Falling in MIMIC-IV, and 1.73 (1.51–1.99) and 1.67 (1.33–2.11) in eICU-CRD. Prophylactic heparin was associated with a lower subdistribution hazard of 28-day post-landmark mortality in MIMIC-IV (sHR=0.908, 95% CI 0.839–0.983, P = 0.018); in an exploratory interaction analysis, the point estimate was more pronounced at higher predicted probabilities of the Persistently High trajectory, but this pattern was not replicated in eICU-CRD (sHR 0.877, 95% CI 0.744–1.034, P = 0.118).

**Conclusion:**

SIC score trajectories stratify septic patients into three trajectory classes—Persistently High, Rising-then-Falling, and Resolving—each showing a graded association with 28-day mortality. Early prophylactic unfractionated heparin showed an exploratory association with lower post-landmark mortality in MIMIC-IV that was not independently replicated in eICU-CRD. Although these findings do not support treatment recommendations or trajectory-based anticoagulation decisions, they provide a basis for formulating hypotheses for future prospective studies.

## 1 Background

Sepsis is a life-threatening syndrome of organ dysfunction arising from a dysregulated host response to infection (Singer et al., 2016). Despite substantial advances in its clinical management, sepsis remains amongst the leading causes of death worldwide (Rudd et al., 2020). Coagulation dysfunction occurs in nearly all septic patients and is considered an initiating factor in the progression of sepsis to multiple organ dysfunction syndrome (MODS) (Yu et al., 2024). The coagulation status of septic patients may evolve from an initial compensated state to decompensated disseminated intravascular coagulation (DIC). The incidence of DIC amongst septic patients reaches as high as 46.8%, and its onset is associated with a substantial increase in mortality, reaching up to 60% (Lyons et al., 2018; Gong et al., 2025). Timely recognition of evolving coagulation abnormalities and corresponding risk stratification are therefore of considerable clinical importance.

The Sepsis-Induced Coagulopathy (SIC) criteria, proposed in 2017, integrate platelet count, international normalised ratio (INR), and the degree of organ dysfunction to assess coagulation status in sepsis (Iba et al., 2017). Adopted by the International Society on Thrombosis and Haemostasis (ISTH) in 2019, the SIC score has since been widely applied for the early screening of DIC (Iba et al., 2019, Iba et al., 2023). By integrating coagulation and organ-dysfunction dimensions, the SIC score supports standardised assessment of coagulation abnormalities and prognostic characterization in patients with sepsis. Nevertheless, SIC is not a static entity: patients with identical baseline scores may follow markedly divergent clinical courses—persistent deterioration, transient fluctuation, or gradual resolution—such that reliance on a single-timepoint assessment risks obscuring underlying heterogeneity in disease progression. Latent class trajectory models based on repeated measurements offer a means of identifying subpopulations with similar patterns of evolution, providing a novel methodological foundation for characterising the dynamic features of sepsis-associated coagulopathy.

Whether septic patients should receive prophylactic anticoagulation remains a subject of considerable debate (Yatabe et al., 2018). Prevailing opinion holds that prophylactic anticoagulation may substantially reduce the incidence of DIC and improve outcomes in sepsis. However, sepsis is fundamentally a highly heterogeneous clinical syndrome, and the benefit of anticoagulant therapy is likely to vary according to coagulation status, disease severity, and individual biological characteristics (Antcliffe et al., 2025). The repeated failure of single-target anticoagulant therapies in clinical trials further underscores the profound complexity of sepsis pathophysiology and the intrinsic heterogeneity of the patient population (Umemura and Yamakawa, 2016; Pei et al., 2022). Heparin, a widely used anticoagulant in clinical practice, may confer additional anti-inflammatory, endothelial-protective, and immunomodulatory effects beyond its anticoagulant action, lending it considerable therapeutic promise in sepsis (Yu et al., 2024). Prior studies have suggested that early heparin administration is associated with improved outcomes, particularly amongst patients with moderate-to-severe sepsis-induced coagulopathy (Zou et al., 2022; Huang et al., 2023; Ushio et al., 2024), although findings from randomised trials and other studies have not been consistent (Iba et al., 2024; Jaimes et al., 2009). Building on the characterisation of dynamic SIC trajectory classes, further investigation into the association between early heparin use and clinical outcomes may therefore help generate hypotheses for future prospective research.

Accordingly, this study leverages the Medical Information Mart for Intensive Care IV (MIMIC-IV) database and applies latent class mixed modelling to characterise SIC score trajectories over the first seven days following ICU admission (Proust-Lima et al., 2017). Within a 48-hour landmark framework, we additionally conducted hypothesis-generating analyses of the association between early prophylactic unfractionated heparin exposure and subsequent in-hospital mortality.

## 2 Methods

### 2.1 Data source and study population

The derivation cohort in this study was drawn from the MIMIC-IV database (version 3.0; Certification No. 31397927; https://mimic.mit.edu/), a large, publicly available database containing detailed clinical information on patients admitted to the Beth Israel Deaconess Medical Center between 2008 and 2022. The external validation cohort was drawn from the eICU Collaborative Research Database (eICU-CRD, version 2.0; https://eicu-crd.mit.edu/), which encompasses clinical data from multiple ICUs across the United States. Both databases include laboratory results, medication records, vital signs, length of ICU stay, and other ICU-specific clinical information for each patient. All personal data in both databases were de-identified to protect patient privacy, and the present study was therefore exempt from institutional ethical review and informed consent requirements.

Sepsis was defined in accordance with the Sepsis-3 criteria as suspected or confirmed infection accompanied by a Sequential Organ Failure Assessment (SOFA) score ≥2 (Singer et al., 2016). Exclusion criteria comprised: age <18 or >90 years; non-first ICU admission; ICU length of stay <48 hours; pregnancy; and onset of sepsis more than 48 hours after ICU admission. To ensure stability of trajectory modelling, patients with fewer than two available timepoints for SIC score calculation during their ICU stay were further excluded (**Figure 1**).

**Figure 1:**
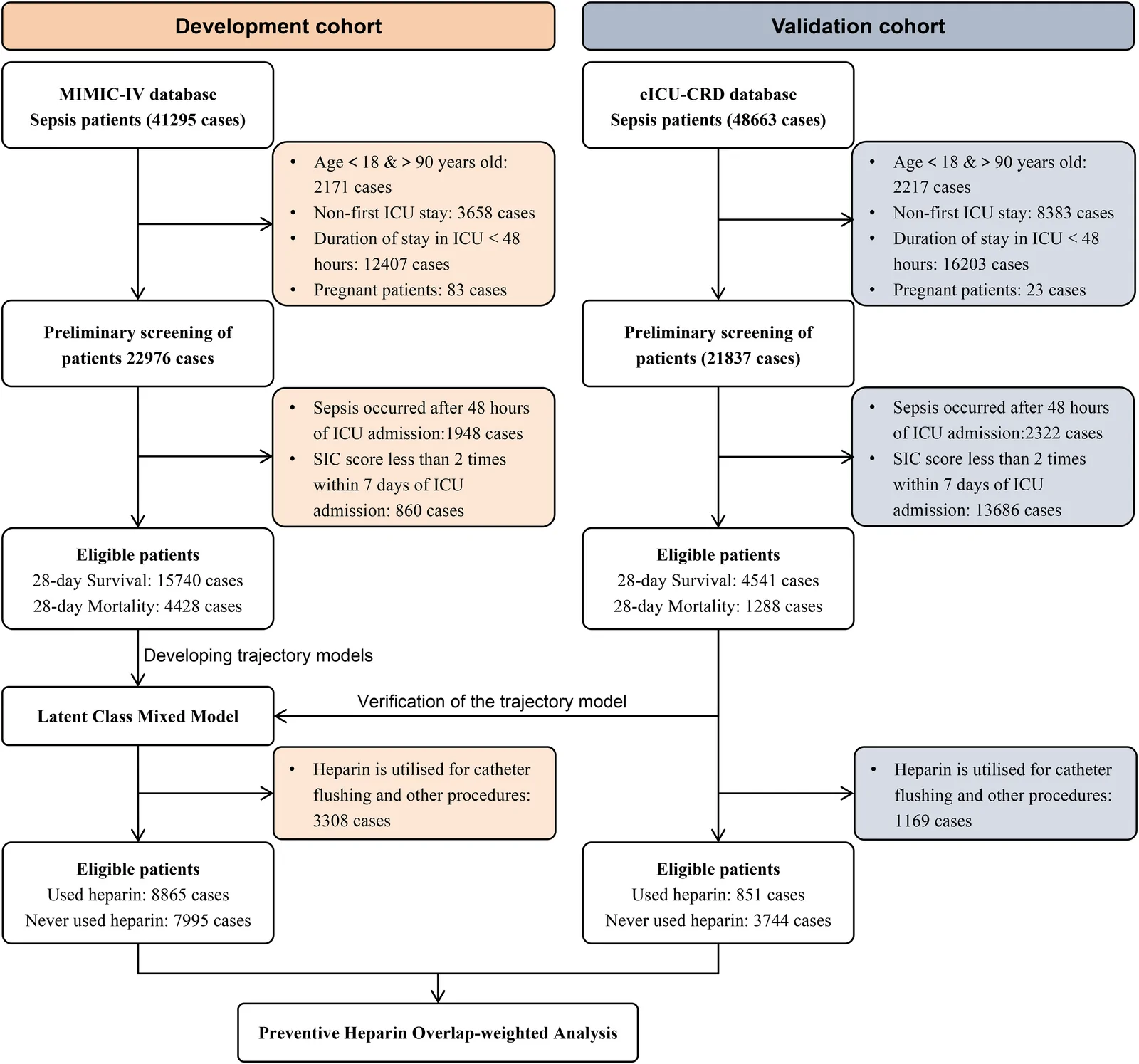
Flowchart of patient inclusion and exclusion for the study. eICU-CRD eICU collaborative research database, MIMIC-IV medical information mart for intensive care IV.

### 2.2 Feature extraction and definitions

For baseline characteristics, we extracted patient demographics, Charlson comorbidities, and the earliest laboratory, scoring, and treatment-related variables recorded within the first 24 hours of ICU admission, including age, sex, race, comorbidities, vital signs, biochemical parameters, complete blood count, coagulation-related tests, Sequential Organ Failure Assessment (SOFA) score, Acute Physiology Score III (APS III), and use of invasive mechanical ventilation. As APS III is unavailable in eICU-CRD, APACHE IV was used as a substitute measure of disease severity in the validation cohort. The SIC score was calculated according to the ISTH criteria from platelet count, INR, and the organ-dysfunction component of SOFA, yielding a total score ranging from 0 to 6; only the highest daily SIC score for each patient was retained. Because the latent class mixed model employed in this study estimates unbalanced longitudinal data via direct maximum likelihood using all available measurements for each patient, no imputation was required for the longitudinal SIC sequences. For baseline characteristics, variables with a missing proportion exceeding 50% were excluded, and missing values for the remaining continuous variables were imputed using the median.

The primary event for the trajectory–outcome analysis was in-hospital mortality within 28 days of ICU admission, with survival to discharge treated as a competing event and administrative censoring applied beyond 28 days. In accordance with target trial emulation principles regarding eligibility, treatment assignment, and alignment of follow-up initiation (Hernán et al., 2016), exposure to unfractionated heparin was assessed exclusively within the first 0–48 hours after ICU admission, with the 48-hour landmark serving as the origin of follow-up, thereby avoiding the misclassification of survival time accrued during the exposure-ascertainment window as treatment-group follow-up time. Patients were classified into a “heparin”group or a “no-heparin” group based on route of administration and dosage; intravenous infusions, line-flushing or catheter/dialysis-circuit maintenance doses, and low-molecular-weight heparin (LMWH) were excluded from this classification. The exposure-ascertainment window spanned the first 48 hours after ICU admission, follow-up began at the 48-hour landmark, and the primary outcome was in-hospital mortality within 28 days post-landmark, with survival to discharge as the competing event.

### 2.3 Research procedures

#### 2.3.1 Identification and external validation of SIC score longitudinal trajectories

Latent class mixed models were fitted using the hlme function in the R package lcmm (version 2.0.2). We fitted Gaussian latent class mixed models with an identity link, treating the bounded 0–6 SIC score as an approximately continuous outcome, with time trends represented by quadratic orthogonal polynomials; both fixed effects and class-specific mean structures incorporated linear and quadratic terms, and random effects were specified as a random intercept and random linear term. Models with one to five latent classes were fitted, with each multi-class model fitted repeatedly using multiple random starting values to mitigate the influence of local optima. The final number of classes was determined by jointly considering log-likelihood, AIC, BIC, SABIC, entropy, class-specific sample proportions, model stability, and clinical interpretability. The stability of the three-class proportions was assessed using 200 patient-level bootstrap replicates. External validation was performed in the eICU-CRD cohort by fixing the time-polynomial basis and all model parameters obtained from the derivation cohort.

The relationship between trajectory class and outcome was illustrated using cumulative incidence functions, with between-group comparisons performed using the Gray test. Fine–Gray subdistribution hazard models were further used to evaluate the association between trajectory class and 28-day in-hospital mortality, using the Resolving trajectory as reference. Model 0 was unadjusted; Model 1 adjusted for age, sex, and Charlson Comorbidity Index (CCI); Model 2 additionally adjusted for SOFA; and Model 3 extended Model 1 by incorporating APS III (MIMIC-IV) or APACHE IV (eICU-CRD). Given that Model 2 employed identical covariates across both cohorts, its results were designated as the primary adjusted comparison, with Model 3 serving as a sensitivity analysis adjusting for database-specific measures of disease severity.

#### 2.3.2 Baseline-feature-based trajectory-class prediction model

Using MIMIC-IV trajectory class as the supervised learning label, multiclass classification models were developed using clinical features first obtained within the first 24 hours of ICU admission. Candidate features with a missing proportion exceeding 50% in the derivation cohort were excluded; for continuous variables with a missing proportion exceeding 20%, corresponding missingness indicator variables were constructed in parallel. Feature selection was performed using a voting procedure across three methods—Lasso, SHAP, and Boruta—within 5-fold cross-validation, with features retained if selected in at least 3 of 5 folds, yielding the final feature set for the model. Multiple algorithms were subsequently compared, including logistic regression, random forest, ExtraTrees, multilayer perceptron, and eXtreme Gradient Boosting (XGBoost), with the latter selected as the final model. To prevent information leakage, feature selection, imputation, and probability calibration were embedded within cross-validation or fitted exclusively on MIMIC-IV; internal predicted probabilities for MIMIC-IV were derived from out-of-fold cross-validation predictions, whilst predictions for eICU-CRD were generated directly from the frozen MIMIC-IV-trained model. Discrimination was assessed using receiver operating characteristic (ROC) curves, calibration was assessed using calibration curves, and feature contributions were interpreted using SHAP values.

#### 2.3.3 Exploratory analysis of early unfractionated heparin use

Exposure to unfractionated heparin was defined as prophylactic heparin administration within the first 48 hours of ICU admission. To evaluate the association between early heparin therapy and 28-day outcomes across SIC trajectory, propensity score overlap weighting (OW) was applied to control for confounding bias. Overlap weighting is a propensity-score-based causal inference method in which each patient is assigned a weight proportional to the probability of belonging to the opposite treatment group (Thomas et al., 2020).

Separate propensity score models were fitted for MIMIC-IV and eICU-CRD. Candidate covariates included age, sex, race, weight, CCI, SOFA, heart rate, mean arterial pressure, platelet count, INR, PTT, white blood cell count, lactate, creatinine, total bilirubin, blood urea nitrogen, haematocrit, red cell distribution width, mean corpuscular volume (MCV), renal disease, liver disease, malignancy, invasive mechanical ventilation, and vasoactive agent use within the first hour of ICU admission. Propensity scores were estimated via logistic regression, with weights of 1−PS assigned to the treatment group and PS to the control group, thereby estimating the average treatment effect in the overlap population. Covariate balance was assessed using the absolute standardised mean difference (SMD), with an absolute SMD <0.10 indicating adequate balance.

The overall association between prophylactic unfractionated heparin and 28-day post-landmark in-hospital mortality was estimated using a weighted Fine–Gray model, with subdistribution hazard ratios (sHR) and 95% confidence intervals reported; weighted cumulative incidence functions and absolute risk differences at days 7, 14, and 28 were also calculated. Heparin weights were incorporated once during expansion of the Fine–Gray dataset, and models employed cluster-robust variance at the patient level. The XGBoost-predicted probability of the Persistently High SIC trajectory (*p*_PH_) was treated as a continuous variable to explore its interaction with heparin exposure. The proportional (subdistribution) hazards assumption for both the overall association model and the interaction model was assessed using the cox.zph function based on Schoenfeld-type residuals. As an exploratory, non-diagnostic safety proxy signal, we constructed a variable capturing new post-landmark transfusion events (based on blood product records with clearly defined administration times), estimated using the same overlap weighting, landmark follow-up origin, and Fine–Gray competing-risks framework as the primary outcome.

### 2.4 Statistical analysis

Data extraction was performed using PostgreSQL (version 17.0). Data analysis and model construction were conducted in R (version 4.4.2) and Python (version 3.9.16). Categorical variables are presented as frequencies and percentages, with between-group comparisons performed using the χ² test or Fisher’s exact test, as appropriate. Non-normally distributed continuous variables are presented as median and interquartile range (IQR), with between-group differences assessed using the Kruskal–Wallis test. All statistical tests were two-tailed, with *P* < 0.05 considered statistically significant.

## 3 Results

### 3.1 Baseline characteristics of survivors and non-survivors

A total of 20,168 and 5,829 patients were ultimately enrolled from MIMIC-IV and eICU-CRD, respectively (**Supplementary Tables S1**, **S2**). Day-by-day missingness rates for SIC scores in both datasets are presented in **Supplementary Table S3**. Amongst the MIMIC-IV cohort, 15,740 patients survived and 4,428 did not; the overall median age was 66 years (IQR, 56–76), with non-survivors (70 years, IQR [60–80]) being significantly older than survivors (65 years, IQR [55–75]; *P* < 0.001). Overall, non-survivors exhibited a substantially greater comorbidity burden than survivors, with median CCI scores of 7 (IQR, 5–9) versus 5 (IQR, 3–7), respectively. The prevalence of liver disease, renal disease, chronic pulmonary disease, myocardial infarction, congestive heart failure, and cerebrovascular disease was significantly higher amongst non-survivors (*P* < 0.001 for all). Baseline platelet count, INR, and PT differed significantly between survivors and non-survivors (*P* < 0.001 for all), with median values of 186×10^9^/L versus 182×10^9^/L, 1.3 versus 1.4, and 14.6 s versus 15.8 s, respectively. The proportion of patients with SIC ≥4 was higher amongst non-survivors than survivors (89.0% vs. 77.2%, *P* < 0.001). Within eICU-CRD, 1,288 patients died within 28 days. In eICU-CRD, non-survivors were older than survivors (median 68 [IQR 58–77] vs 65 [54–76] years, *P* < 0.001), and the proportion of patients with SIC ≥4 was likewise higher amongst non-survivors than survivors (89.0% vs. 77.2%, *P* < 0.001).

### 3.2 Identification and external validation of SIC score trajectory

Information criteria for the one- to five-class models are summarised in **Table 1**. AIC, BIC, and SABIC decreased progressively with increasing class number, suggesting the possible presence of finer latent structure; however, the additional classes resolved by the four- and five-class models comprised disproportionately small sample proportions, with diminished clinical interpretability and cross-sample stability of trajectory shape. Considering goodness of fit, parsimony, adequacy of class-specific sample size, and clinical interpretability jointly, a three-class model was selected as final. The three trajectory shapes were broadly consistent across both cohorts (**Figures 2A, B**). The Persistently High trajectory remained at elevated SIC levels from day 1 onward, declining only modestly by day 7; the Rising-then-Falling trajectory began at a lower level, peaked around days 3–4, and subsequently declined markedly; and the Resolving trajectory declined progressively from an intermediate level and remained low thereafter. Model classification performance (including mean posterior probabilities) is presented in **Supplementary Table S4**, and individual trajectory distributions are shown in **Supplementary Figure S1**.

**Table 1:** Model-fit indices for latent class mixed models for choosing the best number of profiles.

No. of classes	AIC	BIC	SABIC	Entropy	Log likelihood	Proportion of people (%)				
						Class 1	Class 2	Class 3	Class 4	Class 5
1	231678.6	231734	231711.7	1	-115832.3	100.00				
2	228863.4	228950.5	228915.5	0.54	-114420.7	10.81	89.19			
**3**	**227378.7**	**227497.4**	**227449.7**	**0.57**	**-113674.4**	**31.87**	**10.60**	**57.53**		
4	226113.3	226263.6	226203.2	0.61	-113037.6	35.86	8.04	4.26	51.84	
5	225814.3	225996.3	225923.2	0.66	-112884.2	19.27	38.65	32.13	5.54	4.41

AIC, Akaike information criterion; BIC, Bayesian information criteria; SABIC, sample size adjusted Bayesian information criteria.

Bolded values indicate the three-class model that was selected and used for subsequent analysis.

**Figure 2:**
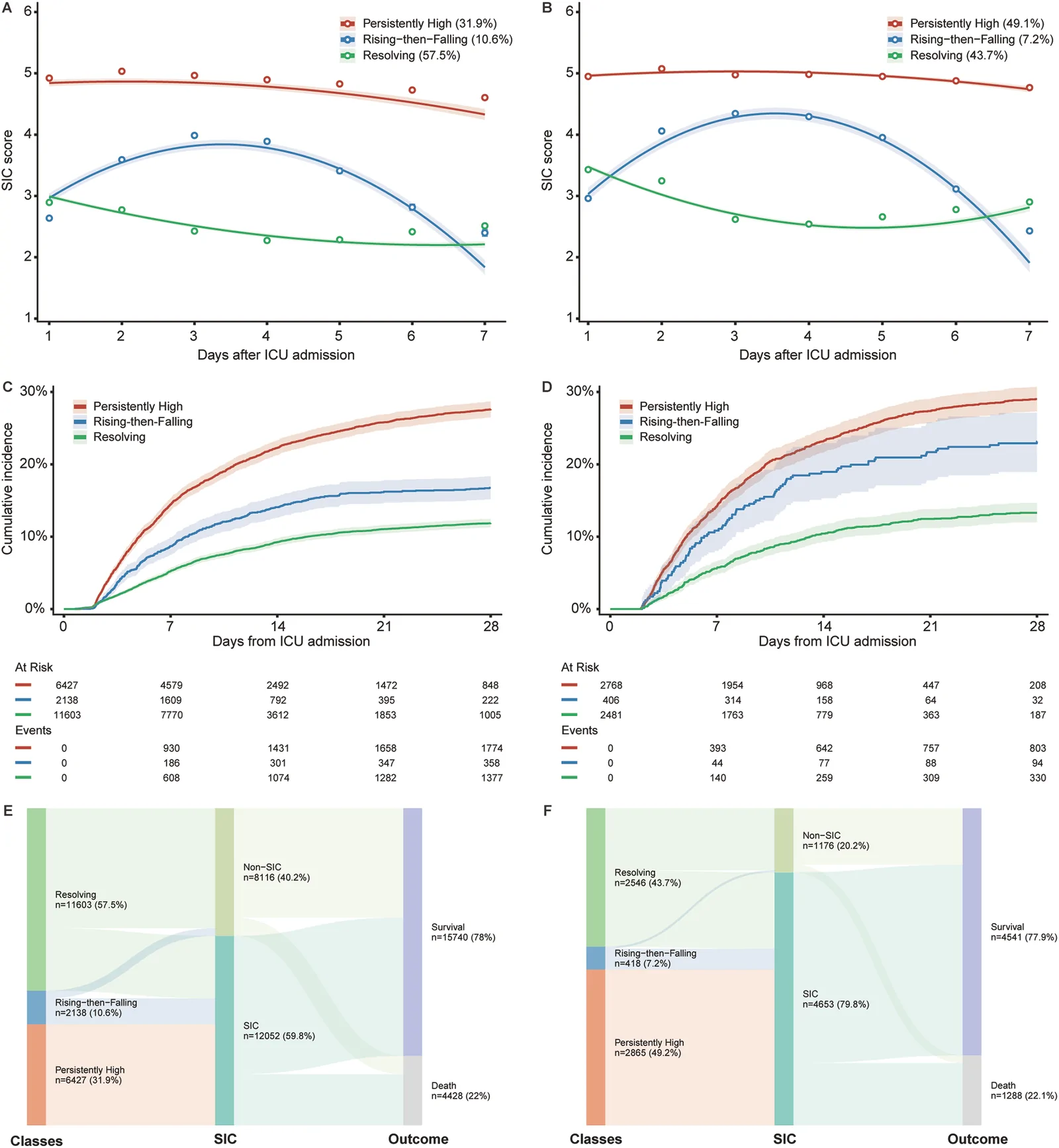
SIC score trajectories, 28-day in-hospital mortality cumulative incidence functions, and trajectory class–SIC status–outcome relationships in MIMIC-IV and eICU-CRD. **(A, B)** The three SIC trajectories in MIMIC-IV and eICU-CRD, respectively. **(C, D)** Cumulative incidence functions for 28-day in-hospital mortality, with survival to discharge treated as a competing event. **(E, F)** Sankey diagrams illustrating the relationships amongst trajectory class, SIC status, and outcome.

Within MIMIC-IV, 6,427 patients (31.9%) were classified as Persistently High, 2,138 (10.6%) as Rising-then-Falling, and 11,603 (57.5%) as Resolving; the corresponding figures in eICU-CRD were 2,865 (49.1%), 418 (7.2%), and 2,546 (43.7%) (**Supplementary Tables S5**, **S6**). Both cohorts demonstrated a greater comorbidity burden, disease severity, and degree of coagulation abnormality in the Persistently High trajectory. In MIMIC-IV, mean SOFA scores for the Persistently High, Rising-then-Falling, and Resolving trajectories were 8.81, 6.08, and 5.50, respectively, and mean APS III scores were 63.4, 52.1, and 48.7; mean platelet counts were 149, 214, and 236×10^9^/L, mean INR values were 2.13, 1.35, and 1.40, and mean total bilirubin concentrations were 4.09, 0.99, and 1.09 mg/dL, with all between-group differences statistically significant (*P* < 0.001). A consistent pattern was observed in eICU-CRD: mean SOFA scores across the three trajectories were 7.96, 5.94, and 5.12, mean APACHE IV scores were 84.3, 78.4, and 70.7, and mean platelet counts were 144, 217, and 244×10^9^/L (*P* < 0.001 for all). **Supplementary Figure S2** presents the distribution of baseline clinical characteristics across the three SIC trajectory classes in MIMIC-IV.

Cumulative incidence curves, with survival to discharge treated as a competing event, indicated 28-day in-hospital mortality proportions of approximately 27.6%, 16.7%, and 11.9% for the Persistently High, Rising-then-Falling, and Resolving trajectories, respectively, in MIMIC-IV, and 28.0%, 22.5%, and 13.0% in eICU-CRD (**Figures 2C, D**). Both cohorts exhibited a consistent gradient, with the highest mortality risk observed in the Persistently High trajectory and the lowest in the Resolving trajectory. Using the Resolving trajectory as reference, unadjusted Fine–Gray models yielded mortality sHR of 2.57 (95% CI: 2.40–2.76) and 1.46 (95% CI: 1.30–1.64) for the Persistently High and Rising-then-Falling trajectories in MIMIC-IV, and 2.41 (95% CI: 2.12–2.74) and 1.85 (95% CI: 1.47–2.33) in eICU-CRD. Following adjustment for age, sex, CCI, and SOFA, these associations attenuated somewhat but remained statistically significant: 1.65 (95% CI: 1.52–1.78) and 1.33 (95% CI: 1.18–1.49) in MIMIC-IV, and 1.73 (95% CI: 1.51–1.99) and 1.67 (95% CI: 1.33–2.11) in eICU-CRD (*P* < 0.001 for all). Results were directionally consistent in models adjusted for database-specific measures of disease severity (**Table 2**), indicating that the association between trajectory class and mortality cannot be fully accounted for by age, comorbidity burden, or early illness severity at ICU admission. Sankey diagrams further illustrate the distribution across trajectory classes, SIC status, and outcome (**Figures 2E, F**).

**Table 2:** Fine–Gray model for the association between SIC score trajectory classes and 28-day in-hospital mortality.

Cohort	Model	Persistently high sHR(95%CI)	P value	Rising-then-falling sHR(95%CI)	P
MIMIC-IV	Model 0	2.57(2.40–2.76)	<0.001	1.46(1.30–1.64)	<0.001
	Model 1	2.34(2.18–2.51)	<0.001	1.43(1.27–1.61)	<0.001
	Model 2	1.65(1.52–1.78)	<0.001	1.33(1.18–1.49)	<0.001
	Model 3	1.75(1.62–1.88)	<0.001	1.33(1.18–1.49)	<0.001
eICU-CRD	Model 0	2.41(2.12–2.74)	<0.001	1.85(1.47–2.33)	<0.001
	Model 1	2.37(2.08–2.69)	<0.001	1.84(1.46–2.31)	<0.001
	Model 2	1.73(1.51–1.99)	<0.001	1.67(1.33–2.11)	<0.001
	Model 3	1.95(1.71–2.23)	<0.001	1.64(1.30–2.06)	<0.001

The Resolving class as a reference. Model 0 is unadjusted. Model 1 adjusted for age, sex, and CCI. Model 2 further adjusted for SOFA. Model 3 adds either the APS III from MIMIC-IV or the APACHE IV from eICU-CRD to Model 1. sHR, Subdistribution hazard ratio.

### 3.3 Prediction of trajectory class from early clinical features

Feature selection across the three methods yielded a final set of 18 features (**Supplementary Figure S3**). Internal AUROC values for the Persistently High trajectory across the five algorithms were as follows: XGBoost, 0.909; multilayer perceptron, 0.903; random forest, 0.901; ExtraTrees, 0.875; and logistic regression, 0.873, with XGBoost demonstrating the best overall performance (**Figure 3A**). The final XGBoost model comprised 400 trees, a maximum depth of 3, a learning rate of 0.05, and a subsample ratio of 0.8. Internal AUROC values for the Persistently High, Rising-then-Falling, and Resolving trajectories were 0.909 (95% CI: 0.905–0.913), 0.695 (95% CI: 0.684–0.705), and 0.824 (95% CI: 0.817–0.830), respectively, with corresponding external AUROC values of 0.835 (95% CI: 0.825–0.845), 0.687 (95% CI: 0.665–0.710), and 0.791 (95% CI: 0.779–0.803) (**Supplementary Table S7**). SHAP analysis identified INR, platelet count, SOFA, RDW, total bilirubin, creatinine, and lactate as key features distinguishing trajectory classes (**Figure 3B**). Calibration slopes ranged from 0.84 to 0.97 internally and from 0.81 to 0.92 externally, indicating generally acceptable in the external cohort (**Figure 3C**; **Supplementary Table S7**). Individual SHAP plots further illustrated the direction and magnitude of each feature’s contribution to class probability across patients (**Supplementary Figure S4**).

**Figure 3:**
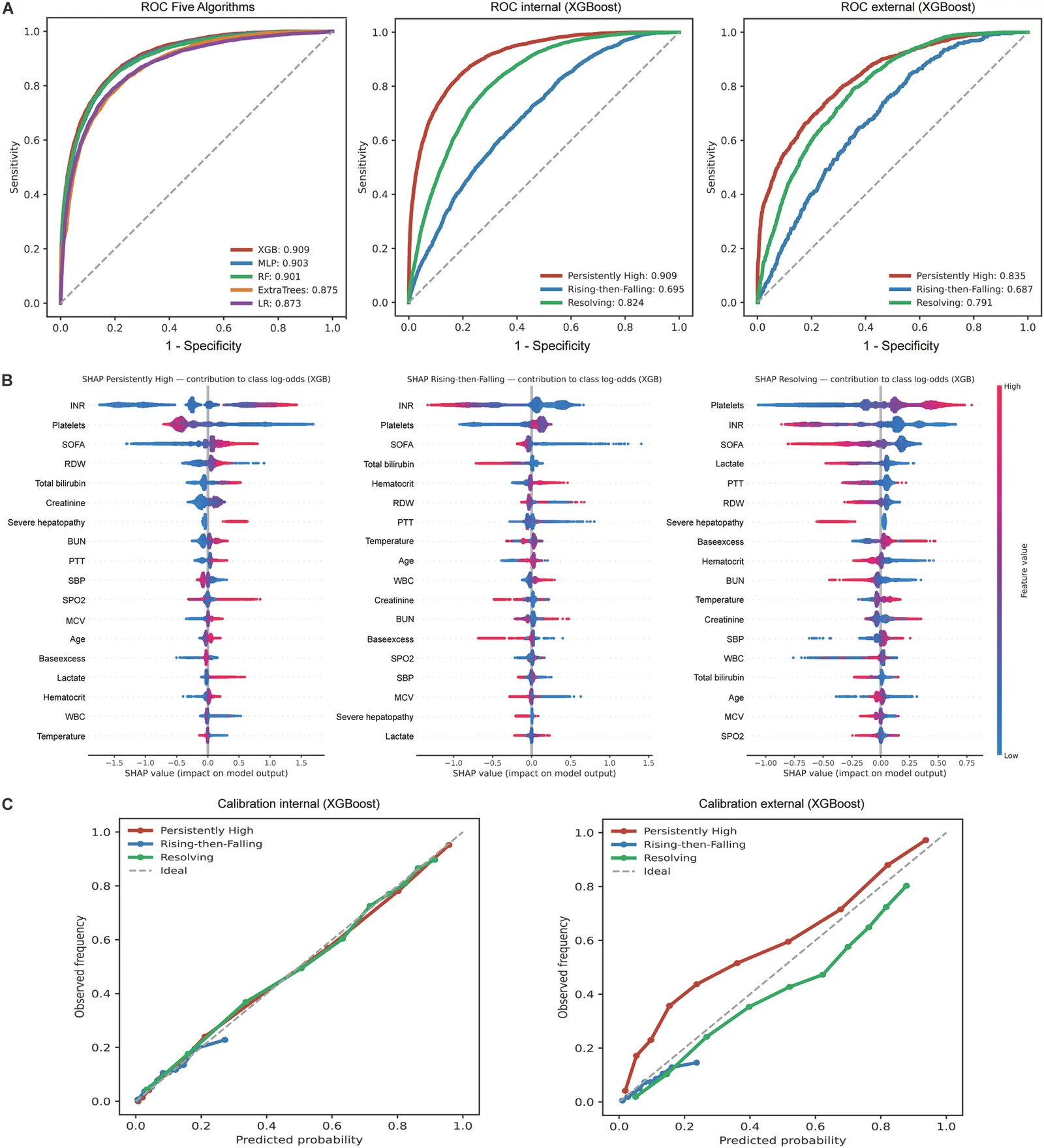
Trajectory class prediction performance across five machine learning algorithms, SHAP interpretation, and calibration for the XGBoost model. **(A)** ROC comparison of the five algorithms for the Persistently High trajectory, together with internal and external ROC curves for XGBoost across the three trajectory classes. **(B)** SHAP beeswarm plots for the three trajectory classes. **(C)** Internal and external calibration curves.

### 3.4 Overlap-weighted analysis of early prophylactic unfractionated heparin and clinical outcomes

Amongst the 20,168 patients in the MIMIC-IV trajectory cohort, 3,308 were excluded owing to intravenous, mixed, or other non-prophylactic heparin administration patterns, yielding a final analytic cohort of 16,860 patients for the prophylactic heparin analysis, comprising 7,995 patients who received no unfractionated heparin and 8,865 who received prophylactic unfractionated heparin only. Of the 5,829 patients in eICU-CRD, 1,234 were excluded, yielding a final analytic cohort of 4,595 patients, comprising 3,744 who received no unfractionated heparin and 851 who received prophylactic unfractionated heparin. Following overlap weighting, the summed weights of the two groups were equal within each cohort. Effective sample sizes for the unexposed and exposed groups were 7,175.4 and 7,927.4 in MIMIC-IV, and 3,043.8 and 840.5 in eICU-CRD, respectively. The maximum post-weighting absolute standardised mean difference remained below the prespecified threshold of 0.10 in both cohorts, indicating adequate balance of covariates included in the propensity score model (**Supplementary Figure S5**; **Supplementary Table S8**).

The proportional subdistribution hazards assumption test for the overall MIMIC-IV model yielded *P* = 0.017, suggesting possible partial violation of this assumption. In the exploratory interaction analysis, the exponentiated heparin-by-*p*_PH_ interaction term was 0.731 (95% CI 0.583–0.915; P = 0.006) in MIMIC-IV (**Supplementary Table S9**), with the heparin-associated sHR trending downward as *p*_PH_ increased (**Figure 4A**). However, the interaction was not replicated in eICU-CRD (sHR=1.279, 95% CI: 0.771–2.121, P = 0.340), and the finding should be regarded as exploratory and hypothesis-generating rather than evidence of an established individualised treatment effect or a basis for trajectory-guided anticoagulation decisions.

**Figure 4:**
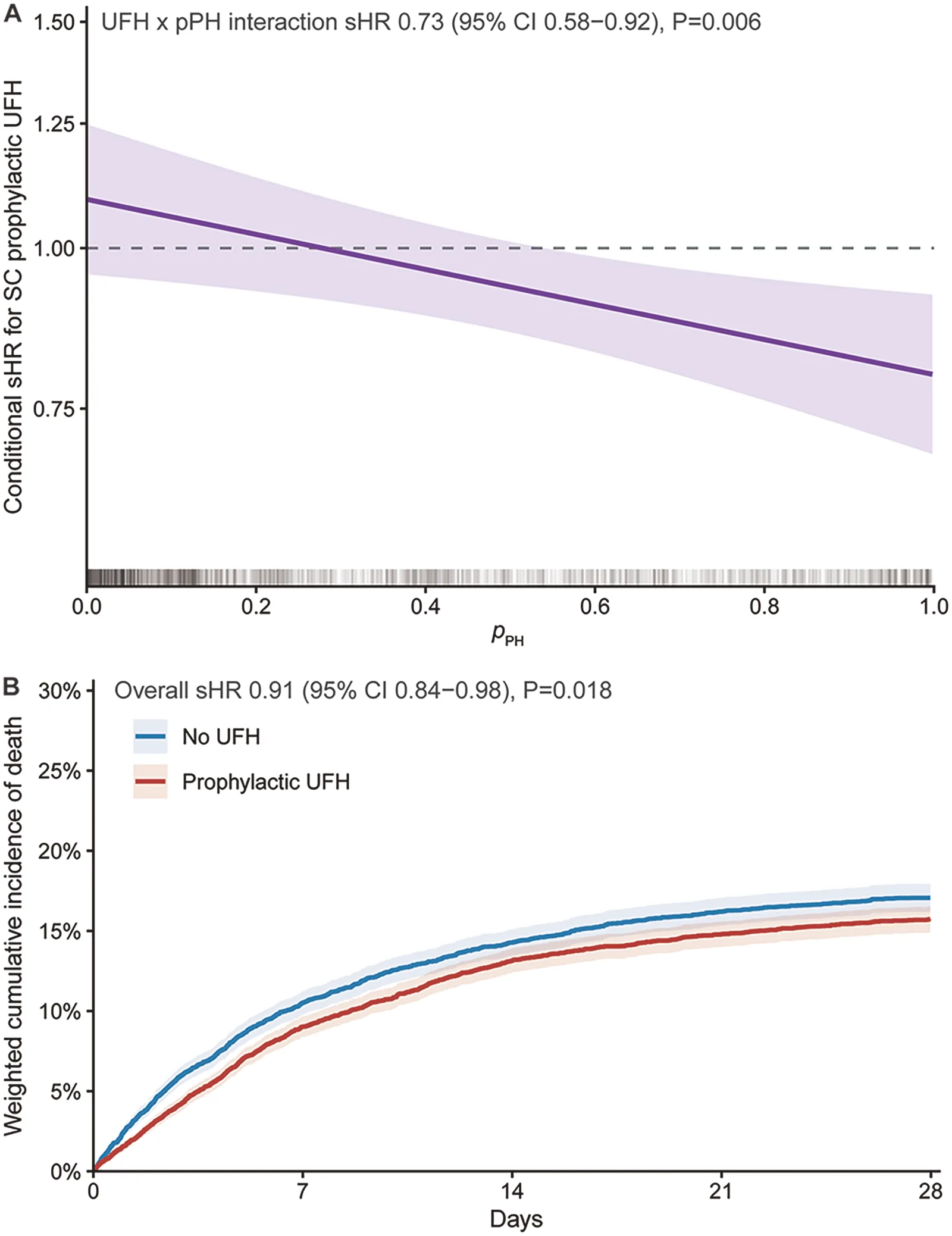
Exploratory overlap-weighted analyses of early prophylactic unfractionated heparin exposure and 28-day post-landmark in-hospital mortality in MIMIC-IV. **(A)** Conditional subdistribution hazard ratio estimates for prophylactic unfractionated heparin exposure across values of the calibrated predicted probability of the Persistently High trajectory (*p*_PH_). The solid line represents the conditional sHR estimate, the shaded area represents the 95% confidence interval, and tick marks indicate the observed distribution of *p*_PH_. **(B)** Overlap-weighted cumulative incidence functions for mortality in the prophylactic unfractionated heparin and no-heparin groups. The overall association was estimated using an overlap-weighted Fine–Gray model. Heparin exposure was assessed during 0–48 hours after ICU admission, and follow-up began at the 48-hour landmark.

In MIMIC-IV, the overlap-weighted Fine–Gray estimate comparing prophylactic unfractionated heparin exposure with no unfractionated heparin exposure was 0.908 (95% CI: 0.839–0.983, *P* = 0.018) (**Figure 4B**; **Supplementary Table S9**). Because the proportional subdistribution hazards test suggested a possible violation for this model (P = 0.017), the sHR should be interpreted as an average association over follow-up rather than as a constant effect. In eICU-CRD, the direction of the effect estimate was consistent with reduced mortality subdistribution risk but did not reach statistical significance (sHR=0.877, 95% CI: 0.744–1.034, *P* = 0.118) (**Supplementary Figure S6**). **Supplementary Figure S7** summarises the sHR and 95% CIs for these primary outcomes in forest-plot format. Weighted cumulative incidence function analysis showed 28-day in-hospital mortality rates of 17.06% in the unexposed group versus 15.72% in the subcutaneous prophylactic heparin group in MIMIC-IV, corresponding to an absolute risk difference of −1.34 percentage points; corresponding cumulative incidence rates in eICU-CRD were 23.65% and 21.10%, respectively, yielding an absolute risk difference of −2.55 percentage points (**Supplementary Table S10**). To explore safety-related outcomes, we constructed a variable capturing new post-landmark transfusion events. Transfusion incidence was lower in the heparin-exposed group than in the unexposed group in both cohorts (MIMIC-IV: sHR=0.83, 95% CI: 0.78–0.89; eICU-CRD: sHR=0.66, 95% CI: 0.52–0.84). Overall, no signal indicating an increased bleeding risk associated with heparin exposure was observed in either cohort. It should be emphasised that transfusion records serve only as an exploratory, non-diagnostic safety proxy and cannot substitute for validated bleeding outcomes, nor can they be used to exclude a potential association between unfractionated heparin and bleeding risk.

## 4 Discussion

In this study, we conducted a longitudinal analysis of SIC scores over the first seven days following ICU admission across MIMIC-IV database, identifying three distinct trajectories—Persistently High, Rising-then-Falling, and Resolving—with a similar trajectory morphology observed in the eICU-CRD external validation cohort. Using the Resolving trajectory as reference, both the Persistently High and Rising-then-Falling trajectories were associated with elevated subdistribution risk of 28-day in-hospital mortality, an association that persisted after adjustment for age, sex, CCI, and SOFA in both cohorts. The subsequently developed XGBoost model demonstrated strong discriminative performance for the Persistently High and Resolving trajectories, though its ability to identify the Rising-then-Falling trajectory was more limited. In the analysis of early unfractionated heparin use, prophylactic heparin showed a statistically significant inverse association with 28-day post-landmark in-hospital mortality in the MIMIC-IV cohort, whereas the eICU-CRD cohort showed a directionally consistent but statistically non-significant association. The interaction between *p*_PH_~ and heparin exposure reached statistical significance in MIMIC-IV, with the point estimate more pronounced at higher predicted probabilities of the Persistently High trajectory. However, this interaction was not replicated in eICU-CRD, and the pattern should be treated as exploratory rather than indicative of a trajectory-dependent treatment effect.

Prior studies have applied machine learning and related approaches to phenotype coagulation abnormalities in septic patients (Zhang et al., 2018; Seymour et al., 2019; Kudo et al., 2021), generally examining the heterogeneity of sepsis-associated coagulopathy from either a cross-sectional phenotypic or a longitudinal perspective. Cai et al. identified three SIC phenotypes amongst 4,993 patients meeting SIC criteria in the MIMIC-IV database using latent class analysis and K-means clustering based on multidimensional clinical and laboratory variables, observing in stratified adjusted analyses that heparin-associated outcomes were concentrated primarily amongst patients with severe coagulopathy and multi-organ dysfunction phenotypes (Cai et al., 2023). However, the class definitions in that study were not based on continuous SIC score trajectories, and heparin exposure encompassed multiple anticoagulant agents; consequently, its class numbering and heparin effect estimates cannot be directly mapped onto those of the present study. Su et al. employed a group-based multi-trajectory model to analyse multiple coagulation indices over the first ten ICU days, similarly identifying three longitudinal coagulation subphenotypes with distinct prognostic risk (Su et al., 2026). Another study applied a joint latent class model (JLCM) to explore the relationship between platelet count and in-hospital mortality in septic patients, demonstrating platelet count to be a key determinant of prognosis (Ye et al., 2024). In comparison with these studies, the present analysis used a daily composite SIC score as the longitudinal indicator, applied a latent class mixed model to characterise score evolution over the first seven days, and additionally treated survival to discharge as a competing event when evaluating the association between trajectory class and in-hospital mortality. Nevertheless, the trajectories identified here reflect longitudinal patterns in the SIC score rather than an established coagulation-biology subphenotype. The machine learning analysis further indicated that early coagulation, organ-function, and inflammatory markers carry meaningful information for trajectory classification: INR, platelet count, and SOFA—core components of the SIC score—also ranked highly in SHAP importance, whilst RDW, total bilirubin, creatinine, and lactate provided complementary information beyond the score itself.

Heparin is amongst the oldest and most widely used anticoagulants globally, possessing, in addition to its well-established anticoagulant properties, diverse immunomodulatory effects and a protective action against glycocalyx shedding. Its anticoagulant effect is mediated through potentiation of antithrombin III activity, alongside inhibition of thrombin generation and thrombin activity (Li and Ma, 2017; Wang et al., 2022). Experimental studies have also reported anticoagulation-independent actions, including interference with HMGB1–LPS interactions, inhibition of cytosolic LPS delivery and caspase-11-dependent inflammatory responses, and possible attenuation of endothelial and glycocalyx injury (Poterucha et al., 2017; Deng et al., 2018; Tang et al., 2021; Yang and Zhang, 2022; Hogwood et al., 2023; Liu et al., 2024). These mechanisms could theoretically be more relevant in patients with persistent coagulation activation and organ dysfunction. However, the present study did not measure thrombin generation, endothelial injury, inflammatory signalling, or other pathway-specific biomarkers. The observed epidemiological associations therefore cannot establish which of these mechanisms were operating.

Despite this therapeutic promise, the clinical application of heparin in sepsis remains subject to debate, and the optimal timing, dosage, and target population for heparin therapy warrant further investigation. A 2024 systematic review by Totoki and colleagues, incorporating three randomised controlled trials comprising 426 patients in total, found that heparin did not significantly reduce mortality risk in septic patients (RR = 0.86, 95% CI: 0.60–1.24), with no significant mortality benefit observed in the subgroup with sepsis-associated DIC (Totoki et al., 2024). Jaimes et al. based on a randomised clinical trial, reported unfractionated heparin to be a potentially safe and feasible intervention in sepsis (Jaimes et al., 2009). A large prospective randomised controlled trial investigating therapeutic-dose heparin anticoagulation in patients with COVID-19 found that treatment-dose heparin increased the probability of survival to hospital discharge (ATTACC Investigators et al., 2021). Zou and colleagues conducted an exploratory analysis of the relationship between heparin therapy and outcomes in septic patients using the MIMIC-IV database, finding that early heparin therapy was associated with reduced in-hospital mortality risk (Zou et al., 2022). Ushio and colleagues found that only septic patients with moderate coagulopathy and SIC scores of 3–4 derived a significant survival benefit from heparin (Ushio et al., 2024), whilst Huang and colleagues similarly reported that heparin therapy primarily reduced ICU mortality amongst patients with an SIC score of 4 (Huang et al., 2023). Overall, owing to the significant heterogeneity of sepsis, heparin therapy may only be beneficial for specific subgroups of patients with sepsis-associated coagulopathy (Tang et al., 2020; Huang et al., 2023; Ushio et al., 2024), potentially reflecting the dual anticoagulant and anti-inflammatory actions of heparin, which may prevent microvascular thrombosis and improve organ perfusion (Beurskens et al., 2020). This study utilised continuous *p*_PH_ to assess whether the association between UFH and *p*_PH_ varies according to the predicted likelihood of a Persistently High clinical course. In the MIMIC-IV dataset, the interaction between UFH and *p*_PH_ was statistically significant (interaction sHR = 0.731, 95% CI: 0.583–0.915), with the point estimate for the UFH–mortality association trending lower as *p*_PH_ increased. However, this interaction was not replicated in the eICU-CRD dataset (interaction sHR = 1.279, 95% CI: 0.771–2.121). Consequently, this interaction in the mimic dataset may reflect genuine effect modification, differences in patient cohorts or clinical practice, residual confounding factors, or statistical instability. This finding does not support using predicted trajectory probability to select patients for prophylactic UFH treatment; it should instead be regarded as hypothesis-generating for future prospective studies specifically designed to test effect modification.

This study has several limitations. First, as a retrospective observational study, the possibility of confounding by indication, unmeasured confounding, and data-recording bias cannot be excluded even with the application of overlap weighting, precluding strict causal inference. Second, this study did not systematically evaluate low-molecular-weight heparin use, cumulative heparin dosing, treatment adherence, or bleeding outcomes. Third, SIC trajectory classes were defined using post-treatment longitudinal information and therefore cannot serve as a stratification variable established at the time of treatment decision-making. Fourth, the three-class model exhibited moderate entropy, and the relatively small sample size of the Rising-then-Falling trajectory limited both the machine learning model’s discriminative performance for this class and its external calibration. Fifth, the 48-hour landmark design excludes patients who were discharged from the hospital or died before the landmark event; thus, its target population is limited to the overlapping cohort of surviving patients who remain at risk, with the endpoint defined as in-hospital death within 28 days after the landmark event – and therefore cannot be directly extrapolated to all sepsis patients. Finally, eICU-CRD was subject to partial missingness in mortality outcomes and laboratory data, and heterogeneity in clinical practice across centres may have influenced the results. Future validation of these findings will require designs anchored to a strict time-zero, reliance on pre-treatment-available features, inclusion of validated bleeding safety outcomes, and prospective study designs.

## 5 Conclusion

This study identified three distinct longitudinal trajectories of SIC score over the first seven days following ICU admission—Persistently High, Rising-then-Falling, and Resolving—with similar trajectory morphology and a consistent prognostic gradient observed in an external validation cohort. An XGBoost model based on early ICU features demonstrated useful discrimination for the Persistently High and Resolving trajectories. In the exploratory overlap-weighted competing-risks analysis, early prophylactic unfractionated heparin exposure was inversely associated with 28-day post-landmark in-hospital mortality in MIMIC-IV, whereas this association was not independently replicated in eICU-CRD. The heparin-related findings should therefore be considered cohort-specific and hypothesis-generating and do not establish a mortality benefit or support the use of SIC trajectory probability to guide anticoagulation decisions. Overall, longitudinal SIC score trajectories may provide a reproducible framework for characterizing SIC heterogeneity and its prognostic associations, but their clinical utility and any treatment-related implications require prospective validation.

## Data Availability

Publicly available datasets were analyzed in this study. Data are fully available at https://eicu-crd.mit.edu/ and https://mimic.mit.edu/.
