# Supplementary material for: Longitudinal sepsis-induced coagulopathy score trajectories and mortality in relation to early prophylactic heparin: a multicentre cohort study
Source: Front Cell Infect Microbiol. 2026 Sep 24;16:1923833. doi: 10.3389/fcimb.2026.1923833 (PMC13645510; doi:10.3389/fcimb.2026.1923833)
Supplement: Supplementary file 1 [file DataSheet1.docx]

**Longitudinal SIC score trajectories and their association with early prophylactic heparin: a multicenter cohort study**

Yuan Cao, Min Wang, Yun Li, Shaoqing Shi, Chengjin Wang, Yiqi Wu, Zhen Gao, Lu Wang, Yuyan Liu, Hongjun Kang

***Supplementary Tables***

*Table S1. Baseline characteristics in survivors and nonsurvivors in the development cohort.*

*Table S2. Baseline characteristics in survivors and nonsurvivors in the validation cohort.*

*Table S3. Daily missing rate of SIC score components in the development (MIMIC-IV) and validation (eICU-CRD) cohorts.*

*Table S4. Comprehensive posterior classification results.*

*Table S5. Baseline characteristics of patients compared across trajectory classes in the development cohort.*

*Table S6. Baseline characteristics of patients compared across trajectory classes in the validation cohort.*

*Table S7. Discriminative performance of the XGBoost model for the three SIC trajectory classes.*

*Table S8. Propensity-score overlap, overlap weights, effective sample size, and covariate balance.*

*Table S9. Full overlap-weighted Fine-Gray model coefficients.*

*Table S10. Descriptive overlap-weighted cumulative incidence of death.*

***Supplementary Figures***

*Figure S1. Daily distribution of observed SIC scores within each of the three SIC trajectory classes.*

*Figure S2. Baseline clinical characteristics across the three SIC trajectory classes in the development cohort.*

*Figure S3. Feature-selection stability across Lasso, SHAP, and Boruta over 5-fold cross-validation.*

*Figure S4. Representative individual-level SHAP force plots for the XGBoost trajectory-class-prediction model.*

*Figure S5. Propensity-score model diagnostics for the overlap-weighted heparin analysis.*

*Figure S6. Overlap-weighted cumulative incidence of 28-day in-hospital death after the 48-hour landmark in eICU-CRD, comparing patients who received prophylactic subcutaneous unfractionated heparin with those who did not.*

*Figure S7. Forest plot summarising the primary mortality association and the exploratory transfusion safety signal (heparin vs none).*

**Definition of the SIC score**

The SIC score comprises a coagulation component and an organ-dysfunction component. The coagulation component consists of two subitems: platelet count (2 points for <100×10⁹/L, 1 point for 100 to <150×10⁹/L, and 0 points for ≥150×10⁹/L) and prothrombin time international normalised ratio (PT-INR; 2 points for >1.4, 1 point for >1.2 and ≤1.4, and 0 points for ≤1.2). The organ-dysfunction component is derived from the sum of SOFA subscores across the four non-coagulation organ systems—respiratory, cardiovascular, hepatic, and renal—mapped to a 0–2 subitem score according to the following rule: 2 points for a summed score ≥2, 1 point for a summed score of 1, and 0 points for a summed score of 0. The three subitem scores are summed to yield the total SIC score, with a total score ≥4 defining SIC. These rules were applied consistently across the MIMIC-IV and eICU-CRD cohorts; scores were calculated hourly, with the maximum value on a given day taken as the representative daily score, and the coagulation-related components entering this calculation using the least favourable value observed within each 24-hour window (i.e., the highest INR and the lowest platelet count).

**Data Sources and Cohort Construction**

The development cohort was derived from the MIMIC-IV database (version 3.0; Certification No.: 31397927; available at <https://mimic.mit.edu/>), a large-scale publicly accessible repository established by the Laboratory for Computational Physiology at the Massachusetts Institute of Technology, encompassing detailed clinical information from patients admitted to the Beth Israel Deaconess Medical Center between 2008 and 2022. The validation cohort was sourced from the eICU Collaborative Research Database (eICU-CRD, version 2.0; available at <https://eicu-crd.mit.edu/>). Data extraction from both databases was performed using PostgreSQL (version 17.0). All statistical analyses and model development were conducted in R (version 4.4.0) and Python (version 3.9.16).

***LCMM model***

The final model parameter information is as follows:

Categorical model with fixed effects (reference category: class3):

| Parameter | Estimated value | SE | P value |
| --- | --- | --- | --- |
| intercept class1 | −0.608 | 0.034 | <0.001 |
| intercept class2 | −1.349 | 0.057 | <0.001 |

Vertical model with fixed effects (separate intercepts, linear terms, and quadratic terms for each category):

| Parameter | Estimated value | SE | P value |
| --- | --- | --- | --- |
| intercept class1 | 4.765 | 0.025 | <0.001 |
| intercept class2 | 3.322 | 0.035 | <0.001 |
| intercept class3 | 2.568 | 0.016 | <0.001 |
| t1 class1 | −39.846 | 4.105 | <0.001 |
| t1 class2 | −53.757 | 5.302 | <0.001 |
| t1 class3 | −80.406 | 2.353 | <0.001 |
| t2 class1 | −21.433 | 1.553 | <0.001 |
| t2 class2 | −149.742 | 3.423 | <0.001 |
| t2 class3 | 26.957 | 1.305 | <0.001 |

Note: op = poly(score_time, 2), t1 = op[, 1], t2 = op[, 2]

Random effects variance-covariance matrix:

| Parameter | Estimated value |
| --- | --- |
| var(intercept) | 0.847 |
| cov(intercept, t1) | 10.428 |
| var(t1) | 18,674.03 |
| Residual standard deviation | 0.501（SE 0.0017） |

Classification Quality:

| Classes | Proportion | Average posterior probability |
| --- | --- | --- |
| Class 1 (Persistently High) | 31.9%（6,427 cases） | 0.756 |
| Class 2 (Rising-then-Falling) | 10.6%（2,138 cases） | 0.706 |
| Class 3 (Resolving) | 57.5%（11,603 cases） | 0.849 |

In the 200 patient-level bootstrap analyses, the medians for the proportions of small, medium, and large categories were 10.4%,31.7%, and 57.9%, respectively; the corresponding 95% empirical confidence intervals were 8.8%–11.9%, 28.0%–33.6%, and 56.1%–62.0%, respectively, indicating that the overall distribution across these three category sizes is stable.

**Propensity Score Overlap Weighting: Technical Details**

***Cohort Definition and Landmark Design***

Patients were classified as receiving prophylactic subcutaneous unfractionated heparin (UFH) if such therapy was documented within 48 hours of ICU admission, using dose-specific criteria (5000 units) cross-validated between prescription/order records and administration records where available; patients with no UFH exposure of any kind (No UFH) served as the comparator. Patients who received intravenous UFH, a mixture of routes, circuit-only (e.g., dialysis-line) heparin, low-molecular-weight heparin, or heparin of undetermined route were excluded from this comparison. A 48-hour landmark was used as the analytic time origin: patients who died or were discharged alive at or before the landmark were excluded, and follow-up for the primary outcome (28-day in-hospital death) was measured from the landmark, with live discharge treated as a competing event.

***Propensity Score Estimation***

Within each cohort (MIMIC-IV and eICU-CRD, modelled separately), the propensity score (PS) for receiving prophylactic subcutaneous UFH was estimated using logistic regression. Candidate covariates were screened for missingness (variables with >50% missing values in either cohort were excluded) and zero variance in either cohort; the resulting common covariate set comprised age, weight, Charlson Comorbidity Index, baseline SOFA score, heart rate, mean blood pressure, blood urea nitrogen, haematocrit, red-cell distribution width, mean corpuscular volume, platelet count, INR, white blood cell count, lactate, creatinine, total bilirubin, chronic renal disease, chronic liver disease, malignancy, invasive mechanical ventilation, vasopressor use within 1 hour, sex, and a four-level race/ethnicity variable. Missing covariate values were imputed using the cohort median (continuous variables) or mode (categorical variables).

***Overlap Weight Construction***

Overlap weighting (OW) was applied within each cohort to balance baseline covariates between UFH-treated and untreated patients, targeting the average treatment effect in the overlap population (ATO). Treated patients were assigned a weight equal to (1 − PS) and untreated patients a weight equal to PS, concentrating the pseudo-population around the region of clinical equipoise (PS ≈ 0.5). Covariate balance after weighting was assessed using absolute standardised mean differences (SMD), with SMD < 0.10 taken as evidence of adequate balance (Figure S5A); propensity-score overlap was inspected graphically (Figure S5B).

***Exploratory Safety Signals***

Given that diagnostic codes for safety outcomes such as bleeding and heparin-induced thrombocytopenia (HIT) in both MIMIC-IV and eICU-CRD lack in-hospital event timestamps—precluding reliable determination of the temporal relationship between diagnostic recording and heparin exposure—this study did not construct safety outcomes from diagnostic codes. Instead, we derived a variable capturing new post-landmark transfusion events from primary records bearing precise timestamps, based on blood product administration records (red blood cells, plasma, platelets, cryoprecipitate, etc.) with clearly defined administration times. Transfusion incidence was lower in the heparin-exposed group than in the unexposed group in both cohorts (MIMIC-IV: sHR=0.83, 95% CI: 0.78–0.88; eICU-CRD: sHR=0.66, 95% CI: 0.52–0.84). Overall, no signal indicating an increased bleeding risk associated with heparin exposure was observed in either cohort.

**Supplementary tables and figures are provided as follows:**

Table S1. Baseline characteristics in survivors and nonsurvivors in the development cohort

| **Variable** | **Missing** | **Overall (N=20168)** | **Survival (N=15740)** | **Death (N=4428)** | **P Value** |
| --- | --- | --- | --- | --- | --- |
| Age (years), median (IQR) | 0 | 66 [56, 76] | 65 [55, 75] | 70 [60, 80] | <0.001 |
| Female | 0 | 8385 (41.6) | 6536 (41.5) | 1849 (41.8) | 0.795 |
| **Race, n (%)** | **0** |  |  |  | **<0.001** |
| White |  | 13100 (65.0) | 10394 (66.0) | 2706 (61.1) |  |
| Black |  | 2089 (10.4) | 1658 (10.5) | 431 (9.7) |  |
| Asian |  | 568 (2.8) | 447 (2.8) | 121 (2.7) |  |
| Other |  | 4411 (21.9) | 3241 (20.6) | 1170 (26.4) |  |
| Height (cm), median (IQR) | 31 | 170.00 [163.00, 178.00] | 170.00 [163.00, 178.00] | 170.00 [163.00, 178.00] | <0.001 |
| Weight (kg), median (IQR) | 0.6 | 79.90 [66.80, 95.60] | 80.00 [67.50, 96.60] | 77.00 [65.00, 92.10] | <0.001 |
| **Score** |  |  |  |  |  |
| SOFA, median (IQR) | 0 | 6.00 [4.00, 9.00] | 6.00 [4.00, 8.00] | 8.00 [5.00, 11.00] | <0.001 |
| APSIII, median (IQR) | 0 | 50.00 [38.00, 66.00] | 48.00 [36.00, 61.00] | 63.00 [49.00, 79.00] | <0.001 |
| **Complications** |  |  |  |  |  |
| Charlson Comorbidity Index, median (IQR) | 0 | 5.00 [3.00, 7.00] | 5.00 [3.00, 7.00] | 7.00 [5.00, 9.00] | <0.001 |
| Congestive heart failure, n (%) |  | 7002 (34.7) | 5230 (33.2) | 1772 (40.0) | <0.001 |
| Renal disease, n (%) |  | 5355 (26.6) | 3948 (25.1) | 1407 (31.8) | <0.001 |
| Chronic pulmonary disease, n (%) |  | 5754 (28.5) | 4387 (27.9) | 1367 (30.9) | <0.001 |
| Diabetes, n (%) |  | 6829 (33.9) | 5305 (33.7) | 1524 (34.4) | 0.385 |
| Malignant cancer, n (%) |  | 2808 (13.9) | 1813 (11.5) | 995 (22.5) | <0.001 |
| Severe liver disease, n (%) |  | 1900 (9.4) | 1222 (7.8) | 678 (15.3) | <0.001 |
| Metastatic solid tumor, n (%) |  | 1268 (6.3) | 656 (4.2) | 612 (13.8) | <0.001 |
| **Vital signs** |  |  |  |  |  |
| Temperature (℃), median (IQR) | 4.5 | 36.78 [36.39, 37.17] | 36.78 [36.40, 37.17] | 36.67 [36.33, 37.06] | <0.001 |
| Heart rate (beats/min), median (IQR) | 0 | 90.00 [78.00, 105.00] | 89.00 [78.00, 104.00] | 94.00 [80.00, 109.00] | <0.001 |
| Respiratory rate (beats/min), median (IQR) | 0.1 | 19.00 [16.00, 24.00] | 19.00 [16.00, 23.00] | 21.00 [17.00, 25.00] | <0.001 |
| Systolic blood pressure (mmHg), median (IQR) | 1.8 | 118.00 [103.00, 137.00] | 119.00 [104.00, 137.00] | 117.00 [102.00, 135.00] | <0.001 |
| Diastolic blood pressure (mmHg), median (IQR) | 1.8 | 65.00 [55.00, 77.00] | 65.00 [55.00, 77.00] | 65.00 [55.00, 78.00] | 0.812 |
| Mean arterial pressure (mmHg), median (IQR) | 0.9 | 80.00 [69.00, 93.00] | 81.00 [70.00, 93.00] | 79.00 [69.00, 92.00] | <0.001 |
| Peripheral oxygen saturation (%), median (IQR) | 0 | 98.00 [95.00, 100.00] | 98.00 [95.00, 100.00] | 97.00 [94.00, 100.00] | <0.001 |
| **Laboratory** |  |  |  |  |  |
| Hematocrit (%), median (IQR) | 1.7 | 31.40 [26.70, 36.50] | 31.50 [26.90, 36.50] | 31.00 [26.40, 36.30] | 0.002 |
| Red blood cell distribution width (%), median (IQR) | 3.7 | 14.90 [13.70, 16.80] | 14.70 [13.60, 16.40] | 15.90 [14.40, 18.10] | <0.001 |
| Mean corpuscular hemoglobin concentration (g/dL), median (IQR) | 3.6 | 32.60 [31.50, 33.80] | 32.70 [31.60, 33.90] | 32.20 [31.10, 33.40] | <0.001 |
| Mean corpuscular volume, median (IQR) | 3.6 | 92.00 [87.00, 96.00] | 91.00 [87.00, 96.00] | 93.00 [88.00, 99.00] | <0.001 |
| Platelets (×10⁹/L), median (IQR) | 2.4 | 185.00 [126.00, 260.00] | 186.00 [129.00, 259.00] | 182.00 [111.00, 263.00] | <0.001 |
| International normalized ratio, median (IQR) | 8 | 1.30 [1.20, 1.70] | 1.30 [1.20, 1.60] | 1.40 [1.20, 2.00] | <0.001 |
| Activated partial thromboplastin time (s), median (IQR) | 8.6 | 31.70 [27.60, 39.40] | 31.30 [27.40, 38.20] | 33.90 [28.40, 44.80] | <0.001 |
| White blood cell count (×10⁹/L), median (IQR) | 3.5 | 11.70 [8.10, 16.50] | 11.50 [7.90, 16.10] | 12.50 [8.50, 18.10] | <0.001 |
| Lactate (mmol/L), median (IQR) | 33.3 | 1.70 [1.20, 2.80] | 1.60 [1.10, 2.50] | 2.20 [1.50, 3.80] | <0.001 |
| Creatinine (mg/dL), median (IQR) | 1.8 | 1.10 [0.80, 1.90] | 1.10 [0.80, 1.70] | 1.40 [0.90, 2.40] | <0.001 |
| Total bilirubin (mg/dL), median (IQR) | 45.1 | 0.70 [0.40, 1.80] | 0.70 [0.40, 1.60] | 0.90 [0.40, 2.50] | <0.001 |
| Aspartate aminotransferase (U/L), median (IQR) | 44.4 | 46.00 [25.00, 110.00] | 43.00 [24.00, 98.00] | 56.00 [29.00, 138.00] | <0.001 |
| Alanine aminotransferase (U/L), median (IQR) | 44.9 | 30.00 [17.00, 70.00] | 29.00 [17.00, 66.00] | 33.00 [18.00, 78.00] | <0.001 |
| **Blood gas** |  |  |  |  |  |
| PH, median (IQR) | 16.8 | 7.36 [7.29, 7.42] | 7.37 [7.30, 7.42] | 7.35 [7.26, 7.41] | <0.001 |
| Base excess (mmol/L), median (IQR) | 16.9 | -1.00 [-5.00, 1.00] | 0.00 [-4.00, 1.00] | -2.00 [-6.25, 1.00] | <0.001 |
| Albumin (g/dL), median (IQR) | 71.4 | 3.00 [2.60, 3.50] | 3.10 [2.60, 3.50] | 2.90 [2.40, 3.40] | <0.001 |
| Anion gap (mmol/L), median (IQR) | 4.2 | 15.00 [12.00, 18.00] | 14.00 [12.00, 17.00] | 16.00 [13.00, 19.00] | <0.001 |
| Blood urea nitrogen (mg/dL), median (IQR) | 1.9 | 23.00 [15.00, 40.00] | 21.00 [14.00, 36.00] | 30.00 [19.00, 50.00] | <0.001 |
| Calcium (mg/dL), median (IQR) | 17 | 8.30 [7.70, 8.80] | 8.30 [7.70, 8.80] | 8.30 [7.70, 8.80] | 0.782 |
| Sodium (mmol/L), median (IQR) | 5.1 | 138.00 [135.00, 141.00] | 139.00 [135.00, 141.00] | 138.00 [134.00, 142.00] | <0.001 |
| Chloride (mmol/L), median (IQR) | 1.9 | 104.00 [99.00, 108.00] | 104.00 [100.00, 109.00] | 102.00 [97.00, 107.00] | <0.001 |
| Potassium (mmol/L), median (IQR) | 5.1 | 4.20 [3.70, 4.70] | 4.10 [3.70, 4.60] | 4.30 [3.80, 4.80] | <0.001 |
| Arterial oxygen partial pressure (mmHg), median (IQR) | 16.8 | 100.00 [56.00, 213.00] | 109.00 [61.00, 242.00] | 79.00 [47.00, 139.00] | <0.001 |
| Arterial carbon dioxide partial pressure (mmHg), median (IQR) | 16.9 | 41.00 [36.00, 48.00] | 41.00 [36.00, 48.00] | 41.00 [34.00, 49.00] | 0.015 |
| Invasive mechanical ventilation, n (%) |  | 12360 (61.3) | 9651 (61.3) | 2709 (61.2) | 0.883 |
| **Outcome** |  |  |  |  |  |
| Sepsis-induced coagulopathy, n (%) |  | 12052 (59.8) | 8796 (55.9) | 3256 (73.5) | <0.001 |
| ICU length of stay (days), median (IQR) | 0 | 4.56 [2.97, 8.35] | 4.28 [2.89, 8.04] | 5.39 [3.39, 9.21] | <0.001 |
| Hospital length of stay (days), median (IQR) | 0 | 11.08 [6.92, 18.95] | 11.84 [7.46, 20.69] | 8.79 [4.95, 14.87] | <0.001 |
| Outcome after leaving the ICU, n (%) |  | 2624 (13.0) | 87 (0.6) | 2537 (57.3) | <0.001 |
| **Class** | **0** |  |  |  | **<0.001** |
| Persistently High |  | 6427 (31.9) | 4296 (27.3) | 2131 (48.1) |  |
| Rising-then-Falling |  | 2138 (10.6) | 1678 (10.7) | 460 (10.4) |  |
| Resolving |  | 11603 (57.5) | 9766 (62.0) | 1837 (41.5) |  |

Abbreviations: IQR, interquartile range; SOFA, sequential organ failure assessment; APSⅢ, Acute Physiology Score Ⅲ; ICU, intensive care unit.

Table S2 Baseline characteristics in survivors and nonsurvivors in the validation cohort

| **Variable** | **Missing** | **Overall (N=5829)** | **Survival (N=4541)** | **Death (N=1288)** | **P Value** |
| --- | --- | --- | --- | --- | --- |
| Age (years), median (IQR) | 0 | 66 [55, 76] | 65 [54, 76] | 68 [58, 77] | <0.001 |
| Female, n (%) | 0 | 2580 (44.3) | 2019 (44.5) | 561 (43.6) | 0.585 |
| **Race, n (%)** | **0** |  |  |  | **0.783** |
| White |  | 4535 (77.8) | 3528 (77.7) | 1007 (78.2) |  |
| Black |  | 562 (9.6) | 446 (9.8) | 116 (9.0) |  |
| Asian |  | 100 (1.7) | 74 (1.6) | 26 (2.0) |  |
| Hispanic |  | 263 (4.5) | 207 (4.6) | 56 (4.3) |  |
| Other |  | 369 (6.3) | 286 (6.3) | 83 (6.4) |  |
| Height (cm), median (IQR) | 0.4 | 170.10 [162.60, 177.80] | 170.10 [162.60, 177.80] | 170.00 [162.60, 177.80] | 0.417 |
| Weight (kg), median (IQR) | 1.1 | 81.60 [67.60, 100.00] | 82.50 [68.00, 100.90] | 79.10 [64.90, 98.30] | <0.001 |
| **Score** |  |  |  |  |  |
| SOFA, median (IQR) | 0 | 6.00 [4.00, 9.00] | 6.00 [4.00, 8.00] | 8.00 [5.00, 11.00] | <0.001 |
| APACHE IV, median (IQR) | 9.3 | 75.00 [58.00, 95.00] | 71.00 [56.00, 89.00] | 89.00 [71.00, 112.00] | <0.001 |
| **Complications** |  |  |  |  |  |
| Charlson Comorbidity Index, median (IQR) | 0 | 4.00 [2.00, 6.00] | 4.00 [2.00, 6.00] | 5.00 [3.00, 7.00] | <0.001 |
| Congestive heart failure, n (%) | 0 | 1260 (21.6) | 1006 (22.2) | 254 (19.7) | 0.067 |
| Moderate-to-severe renal disease, n (%) | 0 | 1167 (20.0) | 891 (19.6) | 276 (21.4) | 0.164 |
| Chronic pulmonary disease, n (%) | 0 | 1095 (18.8) | 834 (18.4) | 261 (20.3) | 0.134 |
| Diabetes mellitus, n (%) | 0 | 1942 (33.3) | 1554 (34.2) | 388 (30.1) | 0.007 |
| Malignancy, n (%) | 0 | 900 (15.4) | 634 (14.0) | 266 (20.7) | <0.001 |
| Metastatic solid tumor, n (%) | 0 | 189 (3.2) | 122 (2.7) | 67 (5.2) | <0.001 |
| Moderate-to-severe liver disease, n (%) | 0 | 271 (4.6) | 177 (3.9) | 94 (7.3) | <0.001 |
| **Vital signs** |  |  |  |  |  |
| Temperature (℃), median (IQR) | 3.7 | 36.80 [36.40, 37.31] | 36.80 [36.40, 37.40] | 36.70 [36.20, 37.27] | <0.001 |
| Heart rate (beats/min), median (IQR) | 7.5 | 97.00 [82.00, 113.00] | 97.00 [81.00, 112.00] | 100.00 [83.00, 115.00] | 0.016 |
| Respiratory rate (beats/min), median (IQR) | 8.7 | 21.00 [17.00, 26.00] | 21.00 [17.00, 26.00] | 22.00 [18.00, 27.00] | 0.003 |
| Systolic blood pressure (mmHg), median (IQR) | 13.7 | 109.00 [94.00, 128.00] | 110.00 [95.00, 129.00] | 106.00 [91.50, 124.00] | <0.001 |
| Diastolic blood pressure (mmHg), median (IQR) | 13.7 | 61.00 [51.00, 74.00] | 62.00 [51.00, 74.00] | 59.00 [49.00, 72.00] | <0.001 |
| Mean arterial pressure (mmHg), median (IQR) | 13.7 | 74.00 [63.00, 88.00] | 75.00 [63.00, 89.00] | 72.00 [60.00, 84.00] | <0.001 |
| Peripheral oxygen saturation (%), median (IQR) | 21.8 | 97.00 [95.00, 100.00] | 97.00 [95.00, 100.00] | 97.00 [95.00, 100.00] | 0.815 |
| **Laboratory** |  |  |  |  |  |
| Hematocrit (%), median (IQR) | 4.7 | 31.10 [26.60, 36.30] | 31.20 [26.70, 36.23] | 31.00 [26.30, 36.30] | 0.382 |
| Red blood cell distribution width (%), median (IQR) | 9.6 | 15.80 [14.50, 17.70] | 15.70 [14.40, 17.50] | 16.40 [14.80, 18.40] | <0.001 |
| Mean corpuscular hemoglobin concentration (g/dL), median (IQR) | 3.8 | 32.80 [31.70, 33.70] | 32.80 [31.70, 33.70] | 32.70 [31.70, 33.80] | 0.879 |
| Mean corpuscular volume, median (IQR) | 3.8 | 90.90 [86.00, 95.50] | 90.60 [86.00, 95.00] | 91.80 [87.00, 97.00] | <0.001 |
| Platelets (×10⁹/L), median (IQR) | 10 | 177.00 [116.00, 250.00] | 181.00 [120.00, 254.00] | 164.00 [93.25, 235.00] | <0.001 |
| International normalized ratio, median (IQR) | 41.3 | 1.60 [1.30, 2.40] | 1.52 [1.26, 2.30] | 1.70 [1.30, 2.60] | <0.001 |
| Activated partial thromboplastin time (s), median (IQR) | 68 | 35.40 [30.20, 43.88] | 35.00 [30.00, 43.00] | 37.00 [31.45, 46.35] | 0.001 |
| White blood cell count (×10⁹/L), median (IQR) | 10.8 | 12.73 [8.30, 18.70] | 12.60 [8.30, 18.50] | 13.60 [8.45, 19.58] | 0.017 |
| Lactate (mmol/L), median (IQR) | 48.4 | 2.10 [1.30, 3.80] | 2.00 [1.20, 3.40] | 2.60 [1.56, 5.10] | <0.001 |
| Creatinine (mg/dL), median (IQR) | 18.5 | 1.50 [0.94, 2.65] | 1.44 [0.90, 2.60] | 1.67 [1.10, 2.90] | <0.001 |
| Total bilirubin (mg/dL), median (IQR) | 34 | 0.90 [0.50, 1.90] | 0.80 [0.50, 1.70] | 1.00 [0.60, 2.50] | <0.001 |
| Aspartate aminotransferase (U/L), median (IQR) | 33.2 | 47.00 [25.00, 125.00] | 42.00 [23.00, 103.00] | 70.00 [35.00, 195.00] | <0.001 |
| Alanine aminotransferase (U/L), median (IQR) | 33.4 | 33.00 [19.00, 77.00] | 31.00 [18.00, 70.00] | 39.50 [21.00, 109.75] | <0.001 |
| **Blood gas** |  |  |  |  |  |
| PH, median (IQR) | 38.4 | 7.34 [7.26, 7.41] | 7.35 [7.27, 7.42] | 7.33 [7.23, 7.40] | <0.001 |
| Base excess (mmol/L), median (IQR) | 53.7 | -3.00 [-8.00, 1.80] | -2.60 [-7.40, 2.00] | -4.60 [-10.00, 1.00] | <0.001 |
| Albumin (g/dL), median (IQR) | 28.7 | 2.50 [2.00, 2.90] | 2.50 [2.10, 3.00] | 2.30 [1.90, 2.80] | <0.001 |
| Anion gap (mmol/L), median (IQR) | 16.7 | 12.00 [9.00, 15.00] | 11.50 [8.00, 15.00] | 13.00 [9.00, 16.00] | <0.001 |
| Blood urea nitrogen (mg/dL), median (IQR) | 18.6 | 31.00 [19.00, 50.00] | 30.00 [18.00, 49.00] | 35.00 [23.00, 54.75] | <0.001 |
| Calcium (mg/dL), median (IQR) | 20.3 | 8.00 [7.40, 8.50] | 8.00 [7.40, 8.60] | 7.90 [7.30, 8.40] | <0.001 |
| Sodium (mmol/L), median (IQR) | 13.3 | 138.00 [134.00, 141.00] | 138.00 [134.00, 141.00] | 138.00 [134.00, 142.00] | 0.368 |
| Chloride (mmol/L), median (IQR) | 18.1 | 104.00 [100.00, 109.00] | 104.00 [100.00, 109.00] | 104.00 [100.00, 109.00] | 0.647 |
| Potassium (mmol/L), median (IQR) | 11.3 | 4.10 [3.60, 4.60] | 4.00 [3.60, 4.60] | 4.15 [3.60, 4.80] | 0.001 |
| Arterial oxygen partial pressure (mmHg), median (IQR) | 38.3 | 96.00 [72.00, 148.00] | 95.00 [72.00, 146.00] | 98.00 [71.97, 151.93] | 0.319 |
| Arterial carbon dioxide partial pressure (mmHg), median (IQR) | 39.3 | 38.60 [31.80, 47.42] | 38.70 [32.00, 47.00] | 38.00 [30.80, 48.40] | 0.422 |
| Invasive mechanical ventilation, n (%) | 0 | 2922 (50.1) | 2120 (46.7) | 802 (62.3) | <0.001 |
| **Outcome** |  |  |  |  |  |
| Sepsis-induced coagulopathy, n (%) | 0 | 4653 (79.8) | 3507 (77.2) | 1146 (89.0) | <0.001 |
| ICU length of stay (days), median (IQR) | 0 | 5.21 [3.38, 9.08] | 5.13 [3.29, 8.88] | 5.79 [3.63, 9.83] | <0.001 |
| Hospital length of stay (days), median (IQR) | 0 | 10.16 [6.44, 16.72] | 11.15 [7.12, 17.74] | 7.27 [4.28, 12.32] | <0.001 |
| **Class** | **0** |  |  |  | **<0.001** |
| Persistently High |  | 2865 (49.2) | 2022 (44.5) | 843 (65.5) |  |
| Rising-then-Falling |  | 418 (7.2) | 318 (7.0) | 100 (7.8) |  |
| Resolving |  | 2546 (43.7) | 2201 (48.5) | 345 (26.8) |  |

Abbreviations: IQR, interquartile range; SOFA, sequential organ failure assessment; APSⅢ, Acute Physiology Score Ⅲ; ICU, intensive care unit.

Table S3. Daily missing rate of SIC score components in the MIMIC-IV and eICU-CRD cohorts.

| **Day** | **MIMIC-IV** | | | **eICU-CRD** | | |
| --- | --- | --- | --- | --- | --- | --- |
|  | **No. patients** | **SIC socre observed** | **Miss rate** | **No. patients** | **SIC socre observed** | **Miss rate** |
| 1 | 20168 | 19146 | 0.051 | 5829 | 4929 | 0.154 |
| 2 | 20168 | 19690 | 0.024 | 5829 | 4389 | 0.247 |
| 3 | 19597 | 16656 | 0.15 | 5828 | 3811 | 0.346 |
| 4 | 14687 | 12109 | 0.176 | 5820 | 2732 | 0.531 |
| 5 | 11333 | 9188 | 0.189 | 4701 | 2021 | 0.57 |
| 6 | 9029 | 7261 | 0.196 | 3812 | 1546 | 0.594 |
| 7 | 7459 | 5905 | 0.208 | 3080 | 1242 | 0.597 |

Table S4. Comprehensive Posterior Classification Results

| **Analysis Dimension** | **Persistently High** | **Rising-then-Falling** | **Resolving** |
| --- | --- | --- | --- |
| **Patients, n(%)** | **6427(31.87)** | **2138(10.6)** | **11603(57.53)** |
| **Posterior Probability Distribution** |  |  |  |
| To Class 1 | 0.7564 | 0.0891 | 0.1545 |
| To Class 2 | 0.1365 | 0.7058 | 0.1578 |
| To Class 3 | 0.0805 | 0.0707 | 0.8488 |
| **Distribution by Probability Threshold (%)** |  |  |  |
| prob>0.7 | 68.91 | 60.23 | 84.12 |

Table S5. Baseline characteristic of patients compared across trajectory classes in the development cohort

| **Variable** | **Persistently High**  **(N=6427)** | **Rising-then-Falling**  **(N=2138)** | **Resolving**  **(N=11603)** | **P Value** |
| --- | --- | --- | --- | --- |
| Age (years), median (IQR) | 66.00 [55.00, 76.00] | 67.00 [56.00, 77.00] | 67.00 [56.00, 77.00] | 0.031 |
| Female | 2420 (37.7) | 851 (39.8) | 5114 (44.1) | <0.001 |
| Race, n (%) |  |  |  | 0.078 |
| White | 4204 (65.4) | 1364 (63.8) | 7532 (64.9) |  |
| Black | 699 (10.9) | 203 (9.5) | 1187 (10.2) |  |
| Asian | 165 (2.6) | 62 (2.9) | 341 (2.9) |  |
| Other | 1359 (21.1) | 509 (23.8) | 2543 (21.9) |  |
| Height (cm), median (IQR) | 170.00 [163.00, 178.00] | 170.00 [163.00, 178.00] | 170.00 [163.00, 178.00] | <0.001 |
| Weight (kg), median (IQR) | 80.10 [68.00, 96.30] | 79.00 [66.80, 94.80] | 79.40 [66.10, 95.40] | 0.001 |
| Score |  |  |  |  |
| SOFA, median (IQR) | 8.00 [6.00, 11.00] | 6.00 [3.00, 8.00] | 5.00 [3.00, 7.00] | <0.001 |
| APSIII, median (IQR) | 60.00 [47.00, 77.00] | 49.00 [37.00, 64.00] | 46.00 [35.00, 59.00] | <0.001 |
| Complications |  |  |  |  |
| Charlson Comorbidity Index, median (IQR) | 6.00 [4.00, 8.00] | 5.00 [3.00, 7.00] | 5.00 [3.00, 7.00] | <0.001 |
| Congestive heart failure, n (%) | 2565 (39.9) | 781 (36.5) | 3656 (31.5) | <0.001 |
| Renal disease, n (%) | 2149 (33.4) | 543 (25.4) | 2663 (23.0) | <0.001 |
| Chronic pulmonary disease, n (%) | 1718 (26.7) | 582 (27.2) | 3454 (29.8) | <0.001 |
| Malignant cancer, n (%) | 1051 (16.4) | 261 (12.2) | 1496 (12.9) | <0.001 |
| Severe liver disease, n (%) | 1589 (24.7) | 59 (2.8) | 252 (2.2) | <0.001 |
| Metastatic solid tumor, n (%) | 410 (6.4) | 134 (6.3) | 724 (6.2) | 0.933 |
| Vital signs |  |  |  |  |
| Temperature (℃), median (IQR) | 36.72 [36.39, 37.11] | 36.72 [36.39, 37.11] | 36.78 [36.44, 37.20] | <0.001 |
| Heart rate (beats/min), median (IQR) | 93.00 [80.00, 109.00] | 89.00 [77.00, 104.00] | 89.00 [77.00, 104.00] | <0.001 |
| Respiratory rate (beats/min), median (IQR) | 20.00 [16.00, 24.00] | 19.00 [16.00, 23.00] | 19.00 [16.00, 24.00] | <0.001 |
| Systolic blood pressure (mmHg), median (IQR) | 113.00 [99.00, 130.00] | 120.00 [105.00, 138.00] | 121.00 [106.00, 139.00] | <0.001 |
| Diastolic blood pressure (mmHg), median (IQR) | 63.00 [53.00, 75.00] | 66.00 [56.00, 79.00] | 66.00 [56.00, 79.00] | <0.001 |
| Mean arterial pressure (mmHg), median (IQR) | 77.00 [67.00, 89.00] | 82.00 [71.00, 95.00] | 82.00 [71.00, 95.00] | <0.001 |
| Peripheral oxygen saturation (%), median (IQR) | 98.00 [95.00, 100.00] | 98.00 [95.00, 100.00] | 98.00 [95.00, 100.00] | <0.001 |
| Laboratory |  |  |  |  |
| Hematocrit (%), median (IQR) | 29.40 [25.00, 34.60] | 33.20 [28.30, 38.40] | 32.10 [27.60, 36.80] | <0.001 |
| Red blood cell distribution width (%), median (IQR) | 16.10 [14.60, 18.20] | 14.50 [13.40, 16.10] | 14.60 [13.50, 16.10] | <0.001 |
| Mean corpuscular hemoglobin concentration (g/dL), median (IQR) | 32.60 [31.30, 33.70] | 32.60 [31.50, 33.70] | 32.70 [31.50, 33.80] | <0.001 |
| Mean corpuscular volume, median (IQR) | 93.00 [89.00, 99.00] | 92.00 [87.00, 96.00] | 91.00 [87.00, 95.00] | <0.001 |
| Platelets (×10⁹/L), median (IQR) | 123.00 [79.00, 190.00] | 193.00 [150.00, 251.00] | 212.00 [157.00, 286.00] | <0.001 |
| International normalized ratio, median (IQR) | 1.70 [1.40, 2.30] | 1.20 [1.10, 1.40] | 1.20 [1.10, 1.40] | <0.001 |
| Activated partial thromboplastin time (s), median (IQR) | 36.40 [31.00, 46.50] | 30.40 [26.80, 37.30] | 30.10 [26.70, 35.50] | <0.001 |
| White blood cell count (×10⁹/L), median (IQR) | 11.00 [7.00, 16.60] | 12.00 [8.50, 16.90] | 12.00 [8.50, 16.40] | <0.001 |
| Lactate (mmol/L), median (IQR) | 2.10 [1.40, 3.70] | 1.80 [1.20, 2.80] | 1.50 [1.10, 2.30] | <0.001 |
| Creatinine (mg/dL), median (IQR) | 1.50 [0.90, 2.50] | 1.10 [0.80, 1.60] | 1.00 [0.70, 1.60] | <0.001 |
| Total bilirubin (mg/dL), median (IQR) | 1.50 [0.70, 4.00] | 0.60 [0.40, 1.00] | 0.60 [0.30, 1.00] | <0.001 |
| Aspartate aminotransferase (U/L), median (IQR) | 64.00 [33.00, 170.00] | 43.00 [24.00, 100.25] | 37.00 [22.00, 77.00] | <0.001 |
| Alanine aminotransferase (U/L), median (IQR) | 36.00 [19.00, 93.00] | 31.00 [17.00, 69.00] | 26.00 [16.00, 58.00] | <0.001 |
| Blood gas |  |  |  |  |
| PH, median (IQR) | 7.36 [7.28, 7.42] | 7.36 [7.29, 7.41] | 7.37 [7.30, 7.42] | <0.001 |
| Base excess (mmol/L), median (IQR) | -2.00 [-7.00, 0.00] | -1.00 [-5.00, 1.00] | 0.00 [-4.00, 2.00] | <0.001 |
| Albumin (g/dL), median (IQR) | 2.90 [2.40, 3.30] | 3.20 [2.70, 3.70] | 3.10 [2.70, 3.50] | <0.001 |
| Anion gap (mmol/L), median (IQR) | 16.00 [13.00, 19.00] | 14.00 [12.00, 18.00] | 14.00 [12.00, 17.00] | <0.001 |
| Blood urea nitrogen (mg/dL), median (IQR) | 30.00 [19.00, 50.00] | 21.00 [15.00, 35.00] | 20.00 [14.00, 34.00] | <0.001 |
| Calcium (mg/dL), median (IQR) | 8.10 [7.60, 8.70] | 8.30 [7.80, 8.90] | 8.30 [7.80, 8.80] | <0.001 |
| Sodium (mmol/L), median (IQR) | 138.00 [134.00, 141.00] | 139.00 [136.00, 141.00] | 139.00 [136.00, 141.00] | <0.001 |
| Chloride (mmol/L), median (IQR) | 103.00 [98.00, 108.00] | 104.00 [100.00, 109.00] | 104.00 [100.00, 108.00] | <0.001 |
| Potassium (mmol/L), median (IQR) | 4.20 [3.70, 4.80] | 4.20 [3.80, 4.60] | 4.10 [3.70, 4.60] | <0.001 |
| Arterial oxygen partial pressure (mmHg), median (IQR) | 89.00 [50.00, 180.00] | 107.00 [60.00, 237.50] | 106.00 [61.00, 227.25] | <0.001 |
| Arterial carbon dioxide partial pressure (mmHg), median (IQR) | 40.00 [34.00, 47.00] | 42.00 [36.00, 49.00] | 42.00 [36.00, 49.00] | <0.001 |
| Invasive mechanical ventilation, n (%) | 3756 (58.4) | 1347 (63.0) | 7257 (62.5) | <0.001 |
| Heparin used, n (%) | 2234 (34.8) | 893 (41.8) | 5775 (49.8) | <0.001 |
| **Outcome** |  |  |  |  |
| Sepsis-induced coagulopathy, n (%) | 6427 (100.0) | 1652 (77.3) | 3973 (34.2) | <0.001 |
| ICU length of stay (days), median (IQR) | 4.87 [3.08, 9.02] | 5.50 [3.52, 8.95] | 4.20 [2.84, 7.83] | <0.001 |
| Hospital length of stay (days), median (IQR) | 12.53 [7.24, 22.08] | 11.98 [7.76, 19.14] | 10.35 [6.68, 17.24] | <0.001 |
| Outcome after leaving the ICU, n (%) | 1337 (20.8) | 278 (13.0) | 1009 (8.7) | <0.001 |
| 28-day mortality, n (%) | 2131 (33.2) | 460 (21.5) | 1837 (15.8) | <0.001 |

Table S6. Baseline characteristic of patients compared across trajectory classes in the validation cohort

| **Variable** | **Persistently High**  **(N=2865)** | **Rising-then-Falling**  **(N=418)** | **Resolving**  **(N=2546)** | **P Value** |
| --- | --- | --- | --- | --- |
| Age (years), median (IQR) | 66.00 [55.00, 76.00] | 67.00 [56.25, 78.00] | 66.00 [55.00, 76.00] | 0.275 |
| Female, n (%) | 1222 (42.7) | 185 (44.3) | 1173 (46.1) | 0.041 |
| **Race, n (%)** |  |  |  | **0.204** |
| White | 2190 (76.4) | 319 (76.3) | 2026 (79.6) |  |
| Black | 283 (9.9) | 40 (9.6) | 239 (9.4) |  |
| Asian | 51 (1.8) | 9 (2.2) | 40 (1.6) |  |
| Hispanic | 143 (5.0) | 21 (5.0) | 99 (3.9) |  |
| Other | 198 (6.9) | 29 (6.9) | 142 (5.6) |  |
| Height (cm), median (IQR) | 170.20 [162.60, 177.80] | 170.10 [162.60, 177.80] | 170.00 [162.50, 177.80] | 0.368 |
| Weight (kg), median (IQR) | 81.20 [67.10, 99.80] | 81.80 [68.18, 98.85] | 82.50 [67.90, 101.30] | 0.187 |
| **Score** |  |  |  |  |
| SOFA, median (IQR) | 8.00 [5.00, 10.00] | 5.00 [3.00, 8.00] | 5.00 [3.00, 7.00] | <0.001 |
| APACHE IV, median (IQR) | 81.00 [64.00, 101.00] | 74.00 [57.75, 95.25] | 68.00 [53.00, 86.00] | <0.001 |
| **Complications** |  |  |  |  |
| Charlson Comorbidity Index, median (IQR) | 4.00 [3.00, 6.00] | 4.00 [2.00, 6.00] | 4.00 [2.00, 6.00] | <0.001 |
| Congestive heart failure, n (%) | 649 (22.7) | 85 (20.3) | 526 (20.7) | 0.166 |
| Moderate-to-severe renal disease, n (%) | 646 (22.5) | 83 (19.9) | 438 (17.2) | <0.001 |
| Chronic pulmonary disease, n (%) | 475 (16.6) | 93 (22.2) | 527 (20.7) | <0.001 |
| Malignancy, n (%) | 478 (16.7) | 58 (13.9) | 364 (14.3) | 0.035 |
| Metastatic solid tumor, n (%) | 99 (3.5) | 8 (1.9) | 82 (3.2) | 0.25 |
| Moderate-to-severe liver disease, n (%) | 216 (7.5) | 9 (2.2) | 46 (1.8) | <0.001 |
| **Vital signs** |  |  |  |  |
| Temperature (℃), median (IQR) | 36.80 [36.30, 37.30] | 36.80 [36.40, 37.30] | 36.80 [36.40, 37.40] | <0.001 |
| Heart rate (beats/min), median (IQR) | 98.00 [81.25, 113.00] | 97.00 [82.00, 110.00] | 97.00 [81.25, 112.00] | 0.684 |
| Respiratory rate (beats/min), median (IQR) | 21.00 [17.00, 26.00] | 21.00 [16.00, 26.00] | 21.00 [17.00, 26.00] | 0.022 |
| Systolic blood pressure (mmHg), median (IQR) | 105.00 [91.00, 123.00] | 111.00 [96.00, 130.00] | 114.00 [98.00, 134.00] | <0.001 |
| Diastolic blood pressure (mmHg), median (IQR) | 59.00 [49.00, 71.00] | 62.00 [51.25, 74.75] | 63.00 [53.00, 76.00] | <0.001 |
| Mean arterial pressure (mmHg), median (IQR) | 71.00 [60.00, 85.00] | 75.00 [63.00, 89.00] | 77.00 [66.00, 91.00] | <0.001 |
| Peripheral oxygen saturation (%), median (IQR) | 97.00 [95.00, 100.00] | 97.00 [94.00, 99.00] | 97.00 [95.00, 100.00] | 0.12 |
| **Laboratory** |  |  |  |  |
| Hematocrit (%), median (IQR) | 30.50 [26.00, 35.60] | 32.70 [27.35, 37.85] | 31.70 [27.30, 36.60] | <0.001 |
| Red blood cell distribution width (%), median (IQR) | 16.30 [14.90, 18.30] | 15.40 [14.10, 17.40] | 15.40 [14.20, 17.20] | <0.001 |
| Mean corpuscular hemoglobin concentration (g/dL), median (IQR) | 32.90 [31.80, 33.90] | 32.55 [31.70, 33.60] | 32.60 [31.70, 33.60] | <0.001 |
| Mean corpuscular volume, median (IQR) | 91.60 [86.80, 96.70] | 90.55 [86.33, 95.00] | 90.00 [86.00, 94.20] | <0.001 |
| Platelets (×10⁹/L), median (IQR) | 124.00 [74.00, 188.00] | 199.00 [161.00, 257.00] | 222.00 [171.00, 293.00] | <0.001 |
| International normalized ratio, median (IQR) | 1.90 [1.50, 2.80] | 1.30 [1.10, 1.50] | 1.40 [1.20, 1.90] | <0.001 |
| Activated partial thromboplastin time (s), median (IQR) | 39.00 [33.38, 47.80] | 31.85 [27.32, 37.30] | 33.10 [28.60, 40.80] | <0.001 |
| White blood cell count (×10⁹/L), median (IQR) | 11.90 [7.00, 18.21] | 13.24 [9.30, 18.70] | 13.47 [9.60, 19.00] | <0.001 |
| Lactate (mmol/L), median (IQR) | 2.60 [1.60, 4.70] | 2.00 [1.40, 3.40] | 1.70 [1.10, 2.70] | <0.001 |
| Creatinine (mg/dL), median (IQR) | 1.81 [1.13, 3.06] | 1.27 [0.90, 2.30] | 1.23 [0.81, 2.16] | <0.001 |
| Total bilirubin (mg/dL), median (IQR) | 1.30 [0.70, 2.90] | 0.70 [0.40, 1.20] | 0.70 [0.40, 1.10] | <0.001 |
| Aspartate aminotransferase (U/L), median (IQR) | 63.00 [30.00, 181.00] | 43.00 [23.00, 95.50] | 36.00 [21.00, 76.25] | <0.001 |
| Alanine aminotransferase (U/L), median (IQR) | 37.00 [21.00, 96.75] | 30.00 [17.00, 70.00] | 28.00 [17.00, 58.00] | <0.001 |
| **Blood gas** |  |  |  |  |
| PH, median (IQR) | 7.33 [7.25, 7.41] | 7.33 [7.24, 7.40] | 7.36 [7.28, 7.42] | <0.001 |
| Base excess (mmol/L), median (IQR) | -4.80 [-9.97, 0.67] | -3.30 [-8.00, 0.92] | -1.00 [-5.70, 3.00] | <0.001 |
| Albumin (g/dL), median (IQR) | 2.40 [2.00, 2.80] | 2.60 [2.10, 3.00] | 2.60 [2.10, 3.00] | <0.001 |
| Anion gap (mmol/L), median (IQR) | 12.60 [9.00, 16.00] | 11.00 [8.10, 14.90] | 11.00 [8.00, 14.00] | <0.001 |
| Blood urea nitrogen (mg/dL), median (IQR) | 36.00 [22.00, 55.00] | 27.00 [18.00, 45.25] | 26.00 [16.00, 43.00] | <0.001 |
| Calcium (mg/dL), median (IQR) | 7.80 [7.20, 8.40] | 8.10 [7.40, 8.60] | 8.10 [7.60, 8.60] | <0.001 |
| Sodium (mmol/L), median (IQR) | 138.00 [134.00, 142.00] | 139.00 [135.00, 142.00] | 137.00 [134.00, 141.00] | <0.001 |
| Chloride (mmol/L), median (IQR) | 104.00 [100.00, 110.00] | 106.00 [101.00, 109.00] | 104.00 [99.25, 108.00] | <0.001 |
| Potassium (mmol/L), median (IQR) | 4.10 [3.60, 4.70] | 4.05 [3.60, 4.70] | 4.00 [3.60, 4.50] | 0.048 |
| Arterial oxygen partial pressure (mmHg), median (IQR) | 96.00 [72.00, 146.00] | 93.35 [69.00, 134.00] | 97.20 [72.15, 156.00] | 0.224 |
| Arterial carbon dioxide partial pressure (mmHg), median (IQR) | 36.40 [29.20, 45.08] | 38.40 [32.60, 49.00] | 41.00 [34.60, 49.00] | <0.001 |
| Invasive mechanical ventilation, n (%) | 1441 (50.3) | 236 (56.5) | 1245 (48.9) | 0.016 |
| Heparin used, n (%) | 352 (12.3) | 67 (16.0) | 442 (17.4) | <0.001 |
|  |  |  |  |  |
| **Outcome** |  |  |  |  |
| Sepsis-induced coagulopathy, n (%) | 2865 (100.0) | 382 (91.4) | 1406 (55.2) | <0.001 |
| ICU length of stay (days), median (IQR) | 5.38 [3.38, 9.54] | 6.25 [4.04, 9.98] | 4.96 [3.22, 8.63] | <0.001 |
| Hospital length of stay (days), median (IQR) | 10.16 [6.11, 17.05] | 11.52 [7.12, 17.43] | 10.03 [6.61, 16.01] | 0.075 |
| Outcome after leaving the hospital, n (%) | 882 (30.8) | 103 (24.6) | 369 (14.5) | <0.001 |
| 28-day mortality, n (%) | 843 (29.4) | 100 (23.9) | 345 (13.6) | <0.001 |

Abbreviations: IQR, interquartile range; SOFA, sequential organ failure assessment; APSⅢ, Acute Physiology Score Ⅲ; ICU, intensive care unit.

Table S7. Discriminative performance of the XGBoost model for the three SIC trajectory classes.

| Cohort | Trajectory category | AUROC | 95%CI | Calibration Slope |
| --- | --- | --- | --- | --- |
| MIMIC-IV | Persistently High | 0.909 | 0.905–0.913 | 0.97 |
|  | Rising-then-Falling | 0.695 | 0.684–0.705 | 0.84 |
|  | Resolving | 0.824 | 0.817–0.830 | 0.94 |
| eICU-CRD | Persistently High | 0.835 | 0.825–0.845 | 0.81 |
|  | Rising-then-Falling | 0.687 | 0.665–0.710 | 0.85 |
|  | Resolving | 0.791 | 0.779–0.803 | 0.92 |

Abbreviation: AUROC, area under the receiver operating characteristic curve.

Table S8. Propensity-score distributions, overlap weights, effective sample sizes, and covariate balance

| Cohort | Group | N | Sum weights | ESS | PS  min / median / max | OW  min / median / max | Max  \|SMD\| |
| --- | --- | --- | --- | --- | --- | --- | --- |
| MIMIC | No UFH | 7995 | 3752.72 | 7175.40 | 0.0015 / 0.4629 / 0.9606 | 0.0015 / 0.4629 / 0.9606 | 0.0237 |
|  | SC UFH | 8865 | 3752.72 | 7927.41 | 0.0004 / 0.5846 / 0.9600 | 0.0399/ 0.4154 / 0.9996 | 0.0237 |
| eICU | No UFH | 3744 | 658.43 | 3043.80 | 0.0003 / 0.1690 / 0.5832 | 0.0003 / 0.1690 / 0.5832 | 0.0019 |
|  | SC UFH | 851 | 658.43 | 840.53 | 0.0041 / 0.2189 / 0.6220 | 0.3780 / 0.7811 / 0.9959 | 0.0019 |

Note. For overlap weighting, control weights equal PS and treated weights equal 1-PS. Equal group sums of weights are expected. ESS=(sum w)^2/sum(w^2). Max |SMD| summarizes measured covariate balance only.

Table S9. Full overlap-weighted Fine-Gray model coefficients

| Cohort | Model | Term interpretation | sHR (95% CI) | P value |
| --- | --- | --- | --- | --- |
| MIMIC | Overall | SC UFH vs no UFH | 0.908 (0.839–0.983) | 0.018 |
|  | Interaction | SC UFH at pPH=0.5 | 0.933 (0.860–1.011) | 0.092 |
|  | Interaction | pPH, no UFH (0 to 1) | 4.544 (3.865–5.343) | <0.001 |
|  | Interaction | UFH x pPH | 0.731 (0.583–0.915) | 0.006 |
| eICU | Overall | SC UFH vs no UFH | 0.877 (0.744–1.034) | 0.118 |
|  | Interaction | SC UFH at pPH=0.5 | 0.903 (0.765–1.066) | 0.229 |
|  | Interaction | pPH, no UFH (0 to 1) | 2.550 (2.038–3.190) | <0.001 |
|  | Interaction | UFH x pPH | 1.279 (0.771–2.121) | 0.340 |

Note. Because *p*_PH__c=*p*_PH_-0.5, the UFH main effect in the interaction model is conditional on *p*_PH_=0.5. The *p*_PH_ main effect refers to untreated patients and a full-scale change from 0 to 1.

Table S10. Descriptive overlap-weighted cumulative incidence of death

| Cohort | Days | No UFH CIF (%) | SC UFH CIF (%) | Risk difference  (percentage points) |
| --- | --- | --- | --- | --- |
| MIMIC | 7 | 10.53 | 9.02 | -1.51 |
|  | 14 | 14.28 | 13.14 | -1.13 |
|  | 28 | 17.06 | 15.72 | -1.34 |
| eICU | 7 | 14.5 | 12.42 | -2.08 |
|  | 14 | 20.27 | 17.47 | -2.8 |
|  | 28 | 23.65 | 21.10 | -2.55 |

Note. Risk differences were calculated as the cumulative incidence in the UFH group minus that in the no-UFH group. Confidence intervals were not estimated; therefore, risk differences should be interpreted as descriptive estimates.

Figure S1. Daily distribution of observed SIC scores within each of the three SIC trajectory classes. Heatmap shading indicates the proportion of patients with each SIC score on each observation day; solid lines represent the fitted trajectory for each class.

Figure S2. Baseline clinical characteristics across the three SIC trajectory classes in the development cohort (MIMIC-IV). The y-axis shows standardised values (Z-scores); points and error bars represent the mean and 95% CI for each class. *P<0.05, ***P<0.001; ns, not significant.

Figure S3. Feature-selection stability across Lasso, SHAP, and Boruta over 5-fold cross-validation. Cell values indicate the number of folds (out of 5) in which each variable was selected by the corresponding method; stars denote the 18 variables retained in the final consensus feature set.

Figure S4. Representative individual-level SHAP force plots for the XGBoost trajectory-class-prediction model. (A) A correctly classified Persistently High case. (B) A correctly classified Resolving case. (C) A true Rising-then-Falling case misclassified as Resolving. Red features increase, and blue features decrease, the predicted probability of the corresponding class.

Figure S5. Propensity-score model diagnostics for the overlap-weighted heparin analysis. (A) Covariate balance (absolute standardised mean differences) before and after overlap weighting in MIMIC-IV and eICU-CRD; the dashed line marks the conventional balance threshold of 0.10. (B) Propensity-score density overlap between patients receiving no unfractionated heparin (No UFH) and those receiving prophylactic subcutaneous unfractionated heparin (Prophylactic UFH) in MIMIC-IV and eICU-CRD.

Figure S6. Overlap-weighted cumulative incidence of 28-day in-hospital death after the 48-hour landmark in eICU-CRD, comparing patients who received prophylactic subcutaneous unfractionated heparin with those who did not. Solid lines show the weighted cumulative incidence; shaded bands show 95% CIs. The overall association was estimated using an overlap-weighted Fine–Gray model treating live discharge as a competing event.

Figure S7. Forest plot summarises the primary mortality association and the exploratory transfusion safety signal (heparin vs none). sHR and 95% CI are from overlap-weighted Fine–Gray models. Primary outcome (28-day mortality): shows the primary association between early prophylactic subcutaneous UFH and 28-day in-hospital mortality after the landmark. Safety (post-landmark transfusion): shows the exploratory association with new transfusion after the landmark, treating live discharge as a competing event and using the same overlap weights, landmark origin, and Fine–Gray framework as the primary analysis.
